# Gain-of-function mutations in *ALPK1* cause an NF-κB-mediated autoinflammatory disease: functional assessment, clinical phenotyping and disease course of patients with ROSAH syndrome

**DOI:** 10.1136/annrheumdis-2022-222629

**Published:** 2022-07-22

**Authors:** Christina Torres Kozycki, Shilpa Kodati, Laryssa Huryn, Hongying Wang, Blake M Warner, Priyam Jani, Dima Hammoud, Mones S Abu-Asab, Yingyos Jittayasothorn, Mary J Mattapallil, Wanxia Li Tsai, Ehsan Ullah, Ping Zhou, Xiaoying Tian, Ariane Soldatos, Niki Moutsopoulos, Marie Kao-Hsieh, Theo Heller, Edward W Cowen, Chyi-Chia Richard Lee, Camilo Toro, Shelley Kalsi, Zohreh Khavandgar, Alan Baer, Margaret Beach, Debra Long Priel, Michele Nehrebecky, Sofia Rosenzweig, Tina Romeo, Natalie Deuitch, Laurie Brenchley, Eileen Pelayo, Wadih Zein, Nida Sen, Alexander H Yang, Gary Farley, David A Sweetser, Lauren Briere, Janine Yang, Fabiano de Oliveira Poswar, Ida Vanessa D Schwartz, Tamires Silva Alves, Perrine Dusser, Isabelle Koné-Paut, Isabelle Touitou, Salah Mohamed Titah, Petrus Martin van Hagen, Rogier T A van Wijck, Peter J van der Spek, Hiromi Yano, Andreas Benneche, Ellen M Apalset, Ragnhild Wivestad Jansson, Rachel R Caspi, Douglas Byron Kuhns, Massimo Gadina, Hidetoshi Takada, Hiroaki Ida, Ryuta Nishikomori, Elena Verrecchia, Eugenio Sangiorgi, Raffaele Manna, Brian P Brooks, Lucia Sobrin, Robert B Hufnagel, David Beck, Feng Shao, Amanda K Ombrello, Ivona Aksentijevich, Daniel L Kastner, Undiagnosed Diseases Network, Maria T. Acosta

**Affiliations:** 1 Inflammatory Disease Section, National Human Genome Research Institute, Bethesda, Maryland, USA; 2 National Institute of Allergy and Infectious Diseases, Bethesda, Maryland, USA; 3 National Eye Institute, Bethesda, Maryland, USA; 4 National Institute of Dental and Craniofacial Research, Bethesda, Maryland, USA; 5 Radiology and Imaging Sciences, National Institutes of Health Clinical Center, Bethesda, Maryland, USA; 6 Section of Histopathology, National Eye Institute, Bethesda, Maryland, USA; 7 National Institute of Arthritis and Musculoskeletal and Skin Diseases, Bethesda, Maryland, USA; 8 Ophthalmic Genetics & Visual Function Branch, National Eye Institute, Bethesda, Maryland, USA; 9 National Institute of Biological Sciences Beijing, Beijing, China; 10 National Institute of Neurological Disorders and Stroke, Bethesda, Maryland, USA; 11 National Institute of Diabetes and Digestive and Kidney Diseases, Bethesda, Maryland, USA; 12 Dermatology Branch, NIH, National Institute of Arthritis and Musculoskeletal and Skin Diseases, Bethesda, Maryland, USA; 13 National Cancer Institute, Bethesda, Maryland, USA; 14 Undiagnosed Diseases Program, Bethesda, Maryland, USA; 15 National Human Genome Research Institute, Bethesda, Maryland, USA; 16 National Heart Lung and Blood Institute, Bethesda, Maryland, USA; 17 Neutrophil Monitoring Laboratory, Applied/Developmental Research Directorate, Frederick National Laboratory for Cancer Research, Frederick, Maryland, USA; 18 Oncogenesis and Development Section, National Human Genome Research Institute, Bethesda, Maryland, USA; 19 Drs. Gilbert and Farley, OD, PC, Colonial Heights, Virginia, USA; 20 Massachusetts General Hospital Center for Genomic Medicine, Boston, Massachusetts, USA; 21 Division of Medical Genetics & Metabolism, Department of Pediatrics, Massachusetts General Hospital, Boston, Massachusetts, USA; 22 Massachusetts Eye and Ear, Boston, Massachusetts, USA; 23 Hospital de Clínicas de Porto Alegre, Porto Alegre, Brazil; 24 Post Graduate Program in Genetics and Molecular Biology, Universidade Federal do Rio Grande do Sul, Porto Alegre, Brazil; 25 Service de Rhumatologie Pédiatrique, Centre de Référence des Maladies Auto-Inflammatoires de l’enfant, Hôpital Bicêtre, AP HP, Université Paris Sud, Bicetre, France; 26 Service de Rhumatologie Pédiatrique, Centre de Référence des Maladies Auto-Inflammatoires et de l’amylose inflammatoire CEREMAIA, Hôpital Bicêtre, AP HP, Université Paris Saclay, Bicetre, France; 27 CeRéMAIA, CHU Montpellier, INSERM, University of Montpellier, Montpellier, France; 28 Hôpital Fondation Adolphe de Rothschild, Paris, France; 29 Depts Internal Medicine and Immunology, Erasmus MC, Rotterdam, The Netherlands; 30 Pathology & Clinical Bioinformatics, Erasmus MC, Rotterdam, The Netherlands; 31 Iizuka Hospital, Iizuka, Japan; 32 Department of Medical Genetics, Haukeland University Hospital, Bergen, Norway; 33 Bergen Group of Epidemiology and Biomarkers in Rheumatic Disease, Department of Rheumatology, Haukeland University Hospital, Bergen, Norway; 34 Department of Ophthalmology, Haukeland University Hospital, Bergen, Norway; 35 Laboratory of Immunology, National Eye Institute, NIH, Bethesda, Maryland, USA; 36 Department of Child Health, University of Tsukuba Faculty of Medicine, Tsukuba, Japan; 37 Division of Respirology, Neurology, and Rheumatology, Department of Medicine, Kurume University School of Medicine, Kurume, Japan; 38 Department of Pediatrics and Child Health, Kurume University School of Medicine, Kurume, Japan; 39 Department of Internal Medicine, Periodic Fevers Research Center, Università Cattolica del Sacro Cuore, Roma, Italy; 40 Dipartimento di scienze dell'invecchiamento, neurologiche, ortopediche e della testa-collo, Fondazione Policlinico Universitario Agostino Gemelli IRCCS, Roma, Italy; 41 Istitute of Genomic di Medicine, Universita Cattolica del Sacro Cuore, Roma, Italy; 42 NYU, New York, New York, USA

**Keywords:** Inflammation, Amyloidosis, Arthritis, Immune System Diseases, Therapeutics

## Abstract

**Objectives:**

To test the hypothesis that ROSAH (retinal dystrophy, optic nerve oedema, splenomegaly, anhidrosis and headache) syndrome, caused by dominant mutation in *ALPK1*, is an autoinflammatory disease.

**Methods:**

This cohort study systematically evaluated 27 patients with ROSAH syndrome for inflammatory features and investigated the effect of *ALPK1* mutations on immune signalling. Clinical, immunologic and radiographical examinations were performed, and 10 patients were empirically initiated on anticytokine therapy and monitored. Exome sequencing was used to identify a new pathogenic variant. Cytokine profiling, transcriptomics, immunoblotting and knock-in mice were used to assess the impact of *ALPK1* mutations on protein function and immune signalling.

**Results:**

The majority of the cohort carried the p.Thr237Met mutation but we also identified a new ROSAH-associated mutation, p.Tyr254Cys.

Nearly all patients exhibited at least one feature consistent with inflammation including recurrent fever, headaches with meningeal enhancement and premature basal ganglia/brainstem mineralisation on MRI, deforming arthritis and AA amyloidosis. However, there was significant phenotypic variation, even within families and some adults lacked functional visual deficits. While anti-TNF and anti-IL-1 therapies suppressed systemic inflammation and improved quality of life, anti-IL-6 (tocilizumab) was the only anticytokine therapy that improved intraocular inflammation (two of two patients).

Patients’ primary samples and in vitro assays with mutated ALPK1 constructs showed immune activation with increased NF-κB signalling, STAT1 phosphorylation and interferon gene expression signature. Knock-in mice with the *Alpk1* T237M mutation exhibited subclinical inflammation.

Clinical features not conventionally attributed to inflammation were also common in the cohort and included short dental roots, enamel defects and decreased salivary flow.

**Conclusion:**

ROSAH syndrome is an autoinflammatory disease caused by gain-of-function mutations in *ALPK1* and some features of disease are amenable to immunomodulatory therapy.

**WHAT IS ALREADY KNOWN ON THIS TOPIC**

- The p.Thr237Met variant in *ALPK1* has been associated with a dominantly inherited form of progressive blindness. ALPK1’s role in human physiology and immune regulation is still under investigation but the protein is known to act as a sensor for bacterial sugars.

**WHAT THIS STUDY ADDS**

- This is the first study to demonstrate that retinal dystrophy, optic nerve oedema, splenomegaly, anhidrosis and headache (ROSAH) syndrome is an autoinflammatory disease caused by gain-of-function mutations in *ALPK1* and it identifies a second *ALPK1* mutation associated with human disease. The study also establishes that ROSAH syndrome can present with a range of systemic features including recurrent fever, uveitis, deforming arthritis, AA amyloidosis, meningeal enhancement and premature mineralisation of the basal ganglia, substantia nigra and red nuclei on MRI and many manifestations of disease are amenable to modulation with anticytokine therapy.

**HOW THIS STUDY MIGHT AFFECT RESEARCH, PRACTICE, OR POLICY**

- This study introduces ROSAH syndrome as a new autoinflammatory disease and emphasises the need for broader awareness of the disease to facilitate early diagnosis so that patients can be evaluated for immunomodulatory treatment before they suffer irreversible damage from chronic inflammation. It also lays the foundation for future studies to investigate the specific impact of IL-6 inhibition on disease course given its success in reducing intraocular inflammation for two patients.

## Introduction

A heterozygous missense variant p.Thr237Met (T237M) in the *alpha kinase 1* gene (*ALPK1*) has been shown to cause a syndrome termed retinal dystrophy, optic nerve oedema, splenomegaly, anhidrosis and headache (ROSAH), denoting the features of ROSAH.^1^ Nevertheless, ALPK1’s role in human biology is still under investigation, and little has been reported about the mechanism through which the *ALPK1* mutation causes ROSAH syndrome.

The initial paper describing families with ROSAH focused on the ophthalmologic manifestations of the disease and proposed that, like many other forms of heritable retinal degeneration, ROSAH syndrome may be a ciliopathy.^2^ In support of this hypothesis, the authors showed that ALPK1 localises to the ciliary basal body in retinal pigment epithelial cells, and primary cilia formation is dysfunctional in primary patient cells.^1^

However, there is increasing evidence that ALPK1 plays a role in innate immune activation.^3–8^ ALPK1 has been shown to act as an intracellular sensor for metabolites produced by a variety of bacteria including *Helicobacter pylori*, *Shigella flexneri* and *Burkholderia cenocepacia*. Specifically, the N-terminal domain of ALPK1 binds bacterial sugars, including ADP-beta-D-manno-heptose (ADP-heptose). On activation, the kinase domain of ALPK1 phosphorylates TRAF-interacting protein with fork head-associated domain, leading to enhanced NF-κB signalling.^3^ Wild-type mice injected with subcutaneous ADP-heptose demonstrated massive neutrophil recruitment and increased production of NF-κB-induced cytokines and chemokines, whereas these responses were compromised in ALPK1 knock-out mice.^3^ ALPK1 has also been linked to inflammatory conditions in humans. Single-nucleotide polymorphisms in *ALPK1* have been associated with an increased risk of gout, while rare variants have been identified in patients with recurrent periodic fevers.^9–11^ These data suggested that patients with ROSAH may have an inflammatory signature. However, systematic analysis of inflammatory features in humans or mice harbouring activating mutations in *ALPK1* has not been performed.

Given ALPK1’s role as an innate immune sensor, we hypothesised that ROSAH syndrome is an autoinflammatory disease. To test this hypothesis, we characterised a large cohort of molecularly diagnosed patients and analysed the effect of *ALPK1* pathogenic variants on protein function and immune signalling.

## Methods

**Figure 1:**
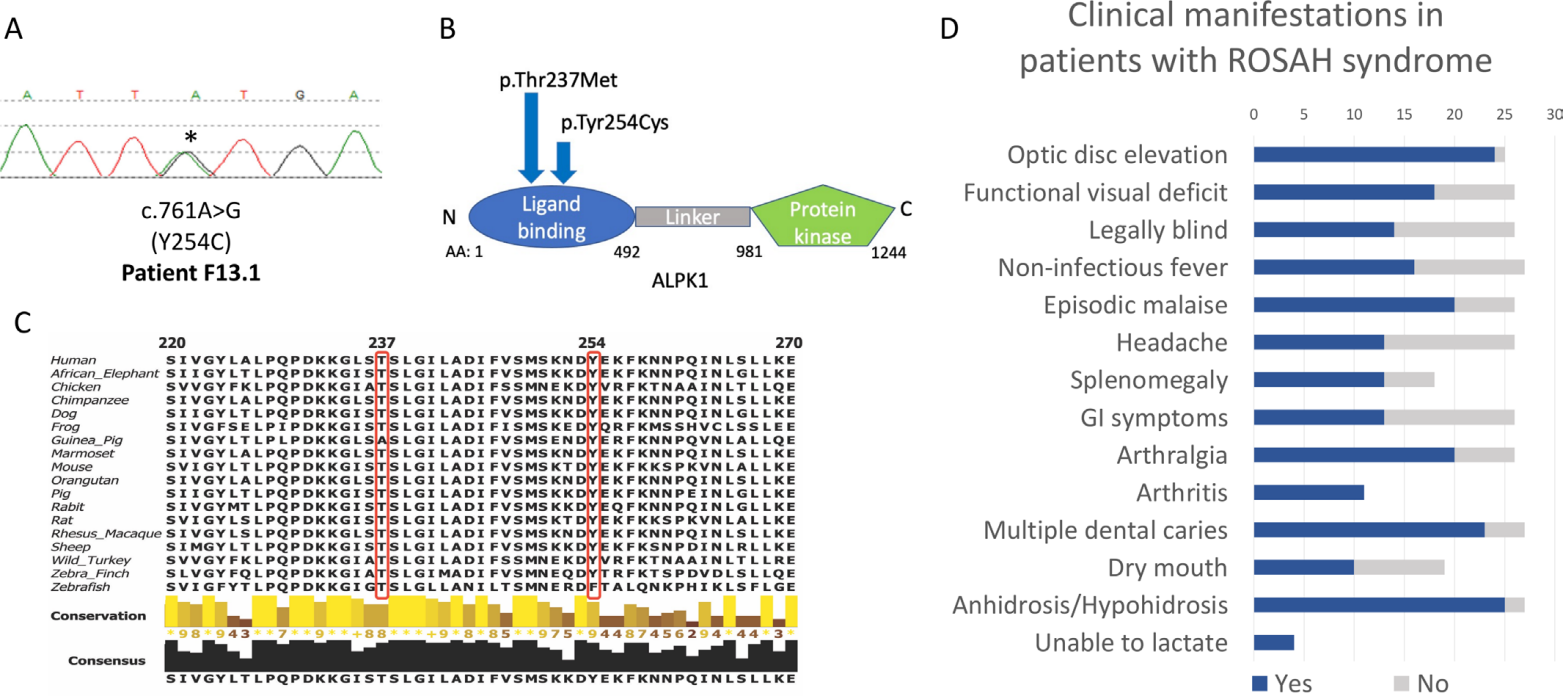
Heterozygous missense mutations of *ALPK1* in the cohort and overview of clinical manifestations observed in patients with ROSAH syndrome. (A) Electropherogram of the previously unreported Y254C mutation for patients F13.1. (B) Domain structure of ALPK1 protein, indicating the location of the observed ROSAH-associated mutations (T237M and Y254C). (C) Schematic showing cross-species conservation of ALPK1 in the regions flanking the T237M and Y254C mutations. Sequences were obtained from Uniprot and multiple sequence alignments were created on Clustal Omega. (D) Bar chart indicating the prevalence of clinical manifestations reported in our ROSAH syndrome cohort. Patient F2.4 has cerebral palsy and was unable to provide any information about subjective clinical features. Blue shading indicates yes, and grey shading indicates no. Splenomegaly as determined by ultrasounds and arthritis as demonstrated by X-ray. Arthritis was present in all individuals evaluated by X-ray. ROSAH, retinal dystrophy, optic nerve oedema, splenomegaly, anhidrosis and headache.

This cohort study included 27 patients with ROSAH syndrome from 8 countries. Twenty patients from 12 unrelated families were confirmed to carry the T237M variant. Six individuals who were a first-degree relative of a proband and had at least two of three features of optic nerve oedema or advanced retinal degeneration, anhidrosis and splenomegaly secondary to red pulp congestion were also included in the cohort. One patient with the ROSAH syndrome phenotype lacked the T237M variant but was found to carry a previously unreported heterozygous missense variant, *ALPK1* p.Tyr254Cys (figure 1A, B).

Between September 2019 and April 2022, information on demographics, clinical manifestations, laboratory parameters and disease course were compiled through interviews of patients or first-degree relatives and review of medical records. During this same period, 11 of these patients were also evaluated at the National Institutes of Health (NIH) Clinical Centre using clinical, radiographic, ultrasonographic and functional examinations. Biological specimens were collected for functional analyses and patients were empirically given immunomodulatory therapies including adalimumab, anakinra, canakinumab and tocilizumab when clinically appropriate and acceptable to the patient.

Additional details are provided in the supplement and include methods for identifying the previously unreported Y254C variant and methods for measurement of soluble biomarkers, gene-expression studies, luciferase assay and development of a knock-in mouse model.

## Results

### Patient population

Twenty-seven patients with ROSAH syndrome were included in this cohort (online supplemental table 1 and figure 1).

10.1136/annrheumdis-2022-222629.supp1 Supplementary data

### Identification of Y254C variant in an individual with ROSAH syndrome

Exome sequencing in patient F13.1 led to identification of a novel heterozygous missense substitution NM_025144.4: c.761A>G; p.Tyr254Cys (Y254C). The patient is of European ancestry, and she is the only clinically affected member in her family. The variant was absent in her mother, and no samples were available from her deceased father or first-degree relatives.

The variant occurs in the ligand binding domain, is predicted to be damaging to protein function by multiple in silico algorithms, including Varsome, PolyPhen-2, SIFT and CADD (24.3) and is absent in the population database gnomAD.^12^ The Tyr254 residue is evolutionarily conserved in vertebrates (figure 1C).

### Clinical features of ROSAH syndrome

**Figure 2:**
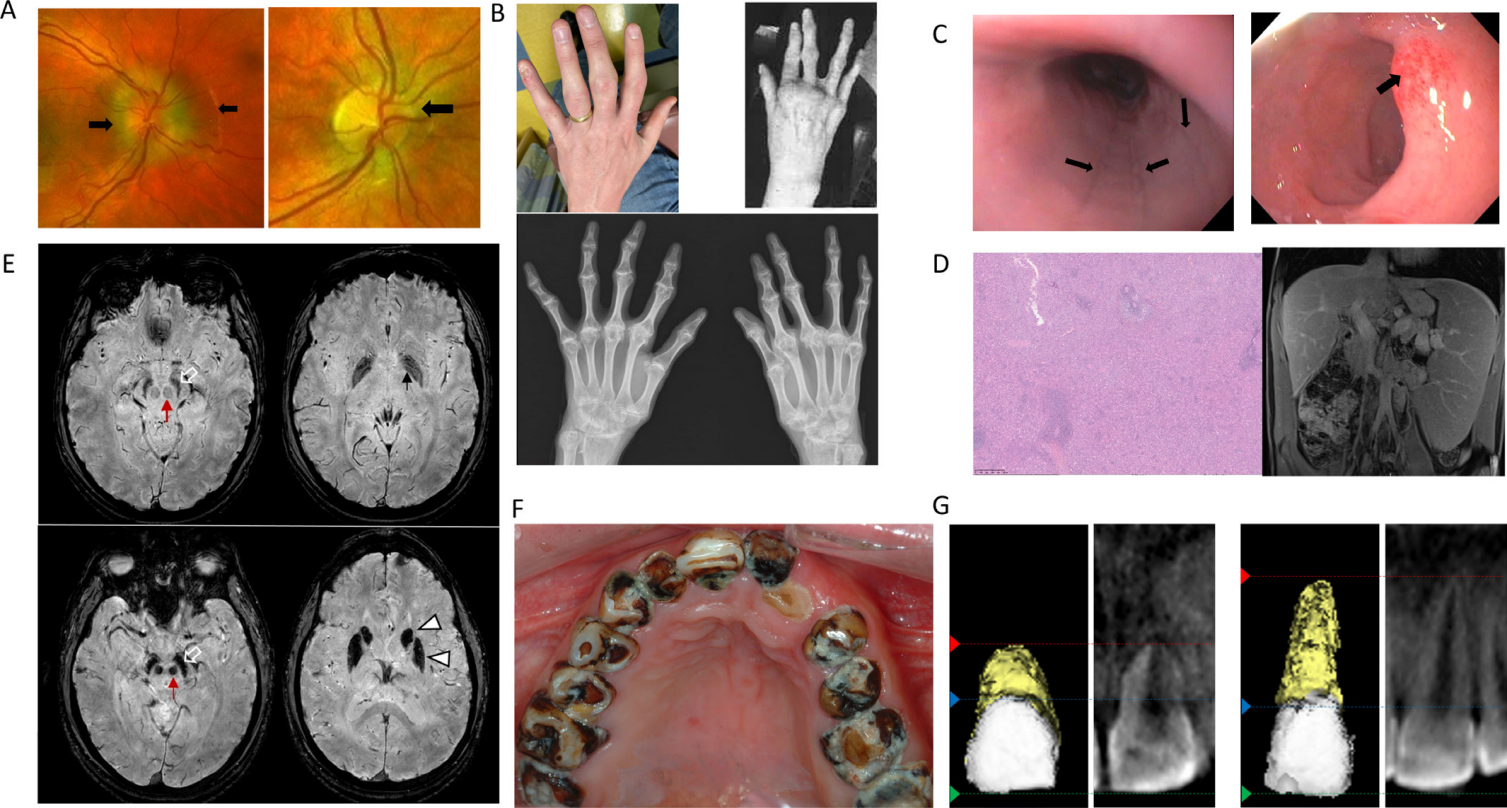
Clinical manifestations associated with ROSAH syndrome. (A) Optic disc elevation. Fundus photographs demonstrating flagrant optic disc oedema (black arrow) in patient F9.2 (left) and more subtle changes in patient F9.3 (right). (B) Inflammatory arthritis with erosive changes in patient F3.3 (top left) and patient F13.1 (top right). X-ray demonstrating advanced diffuse changes of inflammatory arthritis involving wrist, metacarpophalangeal and interphalangeal joints with evolving joint deformities for patient F13.1 (bottom). (C) Gastrointestinal inflammation. Patient F2.2’s endoscopy for dysphagia revealed oesophageal linear furrows (left, arrows) and erythematous duodenal mucosa (right, arrow). (D) Massive splenomegaly. Splenic histology showing red pulp expansion with mild histiocytic hyperplasia from patient F2.2 (resected at age 13, 26×15×6 cm, weighing 1320 grams) (left). Abdominal MRI from patient F1.1 at age 13 demonstrating hepatosplenomegaly with spleen craniocaudal diameter of 22.5 cm and liver craniocaudal diameter of 18.6 cm (right) in the coronal plane (normal range for age: spleen 8–12 cm, liver 8.5–14 cm).^27^ (E) Premature basal ganglia and brainstem mineralisation. Susceptibility weighted imaging from brain MRIs showing decreased signal intensity consistent with premature mineralisation of the globus pallidi (small black arrow), substantia nigra (open white arrows) and red nuclei (red arrows). The mineralisation worsens with age eventually involving the caudate nuclei and putamina (white arrow heads) (top row: patient F1.1, bottom row: patient F7.1). (F) Dental caries. Sjögren’s disease-like pattern of dental caries in patient F4.1. (G) Short dental roots. Three-dimensional rendering and two-dimensional slice of the maxillary central incisor from F5.3 (left) and an age matched healthy control (right). The crown length is similar between the teeth (green line to blue line). However, the root length is one third in F5.3 (blue line to red line). ROSAH, retinal dystrophy, optic nerve oedema, splenomegaly, anhidrosis and headache.

ROSAH patients presented with variable clinical features; however, penetrance was complete in all identified family members. Prominent clinical characteristics are summarised in figure 1D, and representative images are provided in figure 2A–G.

For all families in this cohort, the genetic testing of the proband was prompted by findings on ophthalmologic examination. Ocular manifestations included optic nerve elevation, uveitis, retinal vasculitis and retinal degeneration (online supplemental table 1). For most patients, the initial ophthalmological examination was prompted by subjective visual changes and, in addition to bilateral optic disc elevation, patients were noted to have intraocular inflammation. However, patient F9.1 had no subjective visual symptoms, but bilateral optic disc elevation was observed on a routine health screening examination at age 32. It is also notable that the onset and course of ophthalmological disease varied considerably even within families, and several adults (patients F3.2, F5.2, F9.1, F11.2) lacked subjective visual deficits. The mean age at which patients initially recognised subjective visual deficits was 14.9 years of age (online supplemental figure 2). As an example of the variability in ocular disease, patient F11.3 began experiencing problems with her vision at age 15 and was legally blind by age 21, while her 27-year-old brother (patient F11.2) remains without any significant visual impairment and her 51-year-old mother (patient F11.1) only has decreased night vision. Additionally, patient F5.3 developed retinal detachment leading to left eye blindness by the age of 7, while his 42-year-old father (patient F5.2) remains without any visual deficit. Optic disc elevation was nearly universal in this cohort and was often dramatic but was remarkably subtle in some patients (figure 2A).

Nearly all patients exhibited at least one inflammatory feature which included recurrent fever, malaise, episodic abdominal pain, headaches, transient cytopenias and uveitis with retinal vasculitis. Most patients experienced episodic malaise and many patients experienced non-infectious low-grade fevers. The fever episodes lasted less than 24 hours before resolving spontaneously.

Arthralgia was common (77% (20/26)), with patients reporting involvement of the hands, wrists, elbows, spine, knees, ankles and feet. Nine adults had deforming joint disease that was grossly appreciable on clinical examination or as erosive changes visible on X-ray (figure 2B). In some patients, joint disease was the first clinical manifestation. Patient F12.1 had prominent knee and ankle arthritis by age 4 years, and, after developing debilitating arthritis at age 7, patient F13.1 was evaluated for systemic juvenile idiopathic arthritis (sJIA).

Gastrointestinal symptoms were reported in 14 patients and ranged from episodic abdominal pain, gastro-oesophageal reflux disease (GERD) and dysphagia to constipation and ileus. On endoscopy performed for dysphagia, patient F2.2 was found to have linear oesophageal furrows, gastric erythema and duodenal erosions consistent with ongoing inflammation (figure 2C). Similar erythema of the gastric mucosa was noted by endoscopy in patients F5.2 and F12.1. Most patients evaluated by abdominal ultrasound (72% (13/18)) had splenomegaly, and seven patients underwent splenectomy for abdominal discomfort or cytopenias. Splenic tissue was notable for red pulp expansion (figure 2D, online supplemental table 2). Five patients had hepatomegaly. Transabdominal ultrasound with Doppler imaging, transient liver elastography and abdominal MRI were not consistent with a diagnosis of portal hypertension. Patient F4.1 was found to have microalbuminuria and AA amyloid present on a fat pad biopsy.

Cognitive deficits were rare (4% (1/27)) and only present in patient F2.4 who has cerebral palsy after preterm delivery that was complicated by severe intraventricular haemorrhage. However, recurrent headaches were experienced by many patients (50%, (13/26)) and abnormalities on brain MRIs were common. We reviewed brain MRIs for eight adults who had imagining performed as part of evaluation for headaches or for deep-phenotyping of the cohort. None of the of the patients endorsed extrapyramidal symptoms such as parkinsonism or involuntary movements, although, on clinical examination, patient F7.1 had subtle cog wheel rigidity elicited only with reinforcement manoeuvres. However, seven of the patients had premature mineralisation/calcification of the globus pallidi, red nuclei and substantia nigra, worsening with age, eventually involving the rest of the basal ganglia (figure 2E). White matter abnormalities have also been reported and patient F10.1 initially received a diagnosis of multiple sclerosis after she presented with loss of colour vision and was reported to have multiple lesions on MRI. Focal areas of hyperintensity were noted on fluid-attenuated inversion recovery (FLAIR) in the subcortical white matter of three patients (online supplemental figure 3a). Among the subjects who received post contrast FLAIR imaging (n=8), four showed foci of meningeal enhancement (online supplemental figure 3b), suggesting the possibility of ongoing central nervous system (CNS) inflammation. Additionally, MRI of the orbits showed disc oedema and/or thickening/enhancement of the posterior aspects of the globes in 7 out of 10 subjects, suggesting retinal/choroidal inflammation (online supplemental figure 3c). Two patients had small optic nerves, likely secondary to atrophy, and one subject had chronic retinal detachment.

Dental abnormalities were prevalent. Most patients (85% (23/27)) had multiple dental caries (figure 2F), 5 adults were edentulous, and all seven patients who underwent dental examination at the NIH had some degree of enamel defects with the severity increasing with age. Younger individuals had mild defects in the form of enamel pitting and grooves. Older individuals had severe enamel defects including loss of enamel from the tooth surface. Radiographic examination revealed presence of short and stunted roots in five of the seven patients (online supplemental figure 4a-d). Notably, two of the youngest individuals (both 14 years old) had the shortest roots with root length being less than half of the crown length (figure 2G). Enlargement of the pulp cavity leading to short dental roots in a pattern known as taurodontism was noted in four of seven individuals, with mandibular second molars being the most affected.^13^

**Figure 3:**
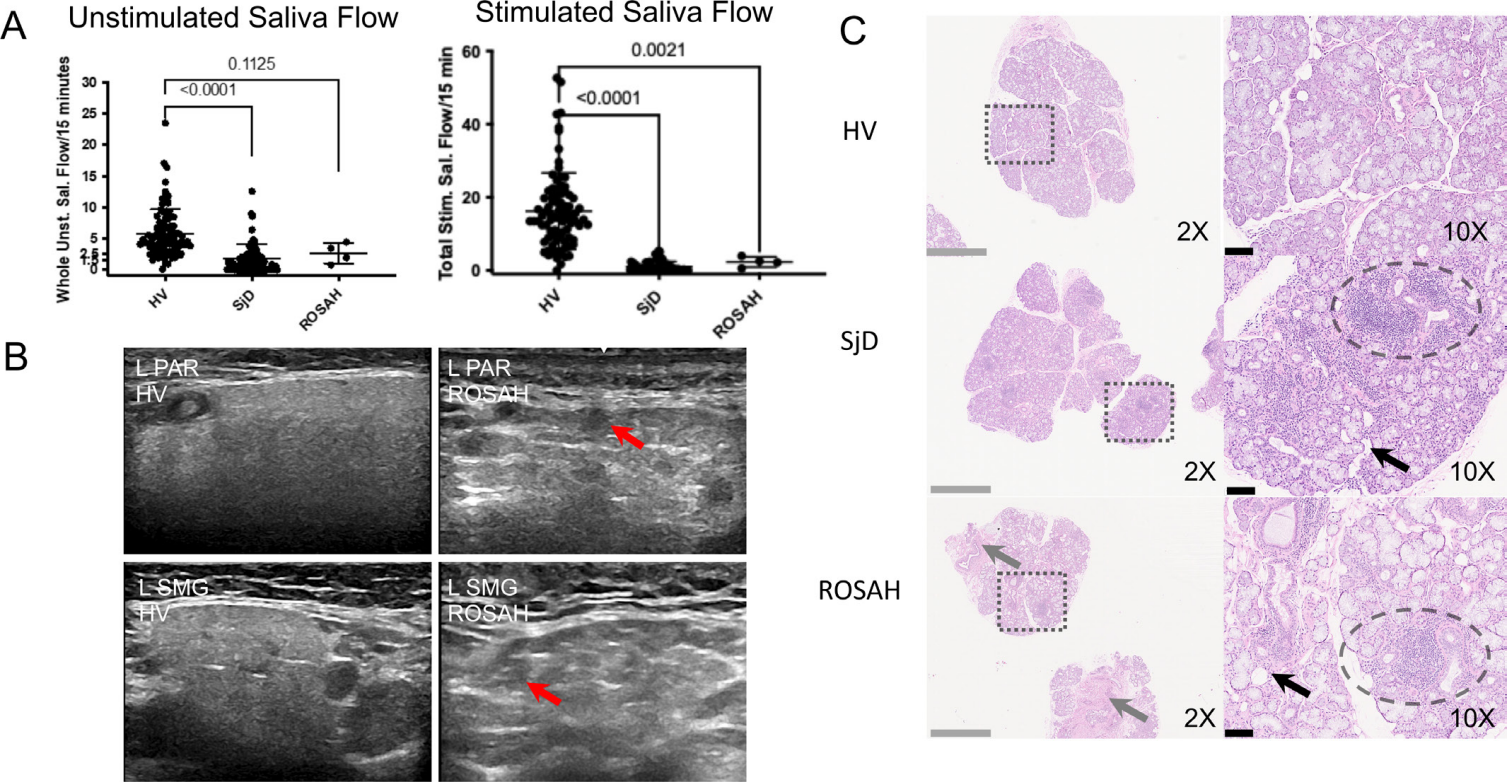
ROSAH patient salivary glands demonstrate salivary hypofunction, altered echoarchitecture and histopathological evidence of inflammation, atrophy and fibrosis. (A) Whole unstimulated saliva flow and total stimulated saliva flow (TSSF, collected while stimulating with 2% citric acid every 30 s) were measured in 4 patients with ROSAH and compared with patients with Sjögren’s disease (SjD) and healthy volunteers (HV). Like SjD, unstimulated and stimulated salivary flow rates were reduced in patients with ROSAH as compared with HV. Statistical significance was only reached for TSSF. (B) The parotid (PAR) and submandibular (SMG) salivary gland ultrasound (SGUS) of ROSAH patients exhibited abnormal echogenicity and homogeneity compared with HV. The most striking finding were isolated (<25% total surface area) to scattered (>50% total surface area) round, hypoechoic lesions which ranged in size from 1.5 mm to 6.5 mm (average of 3–3.5 mm; red arrows). These differ from hypoechoic lesions seen in SjD in shape, size, and distribution and are most likely attributable to pockets of trapped saliva (ie, sialectasias). (C) Labial minor salivary glands (LSG) were inspected using light microscopy. HV LSG are typified by mixed seromucous and mucous acinar cells, and associated ducts, with minimal atrophy or fibrosis and only minimal scattered, typically plasmacytic, inflammation. Alternately, SjD LSG exhibit overall architectural distortion with decreased proportions of seromucous >mucous acinar cells and increased proportion of immune infiltrates (eg, periductal focal lymphocytic sialadenitis (dashed ellipsis) with enhanced diffuse non-sialadenitis). Additional features included: atrophy (eg, decreased lobular size, decreased acinar size), fibrosis (eg, increased interlobular and intralobular collage deposition), adipocyte infiltration (‘fatty infiltration’; black arrow). SMG from patients with ROSAH syndrome exhibit features similar to SjD including periductal focal lymphocytic sialadentis (two of three cases; dashed elipsis), decreased seromucous acinar cells (three of three), prominent increased periductal fibrosis (three of three) and atrophy and increased fatty infiltration (three of three). Additional features included perivascular inflammation, and damage to ducts with mucous extravasation reaction was observed in two cases. ROSAH, retinal dystrophy, optic nerve oedema, splenomegaly, anhidrosis and headache.

Because patients with ROSAH syndrome had a pattern of dental caries that was reminiscent of Sjögren’s Disease (SjD), four patients underwent formal evaluation for SjD (online supplemental table 3).^14 15^ Patients had evidence of hyposalivation with decreased mean unstimulated and stimulated salivary flow (figure 3A). The cause of the decreased stimulated salivary flow in ROSAH syndrome is unclear but ultrasonic imaging of the parotid and submandibular salivary glands was notable for round, hypoechoic lesions that may represent pockets of trapped saliva (figure 3B). Histopathologic analysis of ROSAH labial salivary glands revealed focal inflammation (foci of lymphocytic infiltrate), glandular atrophy with adipocytic replacement and focal mild fibrosis (figure 3C, online supplemental figure 4e). However, subjective eye dryness was not prominent, and features of dry eye disease were not appreciated on slit lamp exam. However, of the 3 patients that had fluorescein ocular staining and Schirmer testing, one individual had an ocular staining score consistent with SjD and one individual had Schirmer testing consistent with SjD.

Dysfunctional production of sweat and breast milk were also common features among patients with ROSAH syndrome. Hypohidrosis or anhidrosis was a nearly universal, present from birth. In this cohort, four parous women were unable to lactate after a total of eight live births.

### Immune system dysregulation

**Figure 4:**
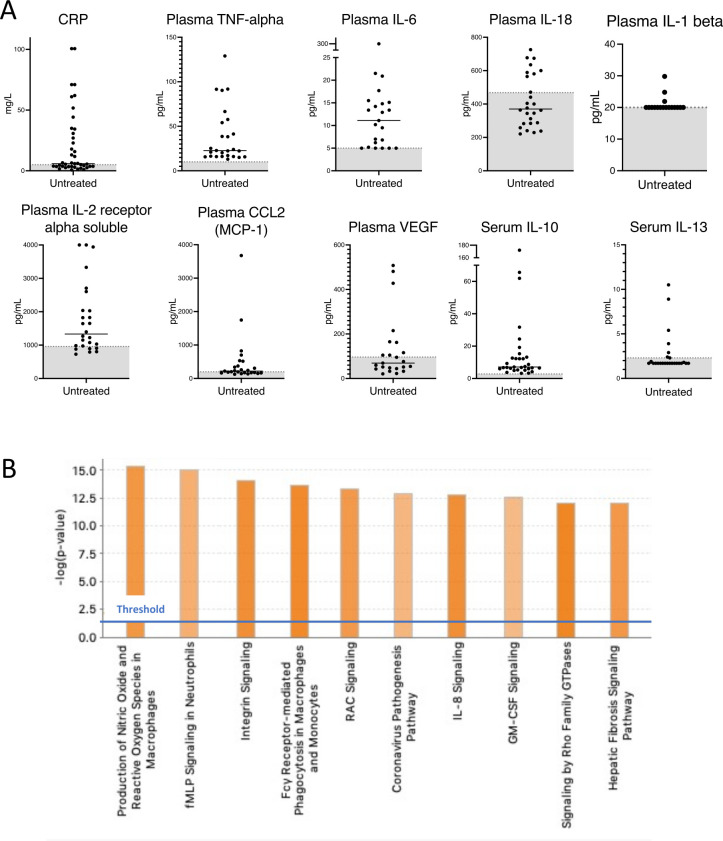
Inflammatory signature in untreated patients with ROSAH syndrome. (A) CRP, cytokine and chemokine levels in serum (n=7) and plasma (n=5) of untreated patients with ROSAH syndrome. Grey zone indicates normal range. (B) Top 10 activated canonical pathways predicted based on differentially expressed genes from whole blood RNA of untreated adults with ROSAH syndrome (n=4) based on Ingenuity Pathway Analysis. Bars denote the different pathways based on Z-scores. CRP, C reactive protein; GM-CSF: granulocyte-macrophage colony-stimulating factor; ROSAH, retinal dystrophy, optic nerve oedema, splenomegaly, anhidrosis and headache.

C reactive protein (CRP) levels were highly variable in untreated patients and significant elevations occurred without change in systemic symptoms and resolved without intervention (figure 4A). For patients with a spleen, episodes of CRP elevation were typically associated with transient cytopenias (online supplemental figure 5a). Seven patients experienced transient neutropenia. Eight individuals underwent bone marrow biopsy for evaluation of cytopenias and there was no evidence of hypocellularity. While cytopenias appeared to improve after splenectomy (online supplemental figure 5b), elevated CRPs were observed after splenectomy in patients F2.2, F11.3 and F12.1 at 101 mg/L, 65 mg/L and 217 mg/L, respectively. Lymphocyte phenotyping by flow cytometry was performed for 12 patients (online supplemental figure 6). Lymphopenia was present in 41% of the patients (5 of 12) without clear predilection for a specific lineage. There was no evidence of increased susceptibility to documented bacterial infections.

Among immune cells, *ALPK1* transcription is highest in neutrophils (online supplemental figure 7a).^16 17^ Therefore, neutrophil function was assessed using assays to evaluate phagosome formation and oxidative burst (online supplemental figure 7b). Based on ingestion of *Staphylococcus aureus* labelled with pH-sensitive dye, neutrophils from one untreated patient (F2.4) demonstrated an early increase in phagocytosis as compared with neutrophils from her affected relatives on cytokine inhibitors and two healthy controls. No difference in phagocytic activity was appreciated in monocytes (online supplemental figure 7c). Dihydrorhodamine flow cytometric assay did not detect any abnormalities in NADPH oxidase activity before or after stimulation with phorbol myristate acetate (PMA).

Pretreatment immunoglobulin levels were normal in most patients (online supplemental figure 8), and consistent with other diseases of autoinflammation, most ROSAH syndrome patients lacked high-titre autoantibodies (online supplemental table 4). Specifically, relevant to the poor dentition observed in patients with ROSAH syndrome, patients had normal IgA levels and lacked anti-SSA and anti-SSB antibodies.

### Inflammatory signature

Untreated patients had recurrent elevations of CRP as well as proinflammatory cytokines and chemokines (figure 4A). Plasma TNF levels were persistently elevated in untreated patients and IL-6, CCL2 (MCP-1), soluble IL-2 receptor alpha and IL-10 were also frequently elevated (online supplemental figure 9a). Patients with ROSAH syndrome also demonstrated elevations of additional cytokines and chemokines including plasma CXCL10 (interferon gamma-induced protein 10 (IP-10)) and serum CXCL1 (GRO-alpha (previously known as neutrophil-activating protein 3)) (online supplemental figure 9b-c). However, no elevations in intracellular IFN-gamma, TNF-alpha or IL-4 were observed after stimulation of peripheral blood cells from two patients (online supplemental table 5).

Analysis of cerebral spinal fluid (CSF) was suggestive of CNS inflammation (online supplemental table 6). CSF neopterin, produced by immune cells after interferon stimulation and shown to correlate with CSF interferon-alpha titres in Aicardi-Goutières syndrome (AGS), was measured in patients F2.2 and F3.4 and found to be elevated.^18–20^ CSF cytology was performed on patient F10.1’s sample and was notable for numerous cells consistent with activated monocytes and lymphocytes. Patient F2.2 had a banked sample available for CSF cytokine analysis and the results were notable for elevation of IL-13 and soluble IL-2 receptor alpha (online supplemental table 7).

RNA extracted from whole blood for four untreated adults with ROSAH had a distinct transcriptomic signature as compared with healthy controls (figure 4B). Many of the differentially expressed genes are involved in innate immune signalling pathways. Production of nitric oxide and reactive oxygen species in macrophages, fMLP signalling in neutrophils, integrin signalling and Fcγ receptor-mediated phagocytosis were among the top upregulated canonical pathways.

### ROSAH syndrome mutations are gain of function and result in enhanced NF-κB signalling

**Figure 5:**
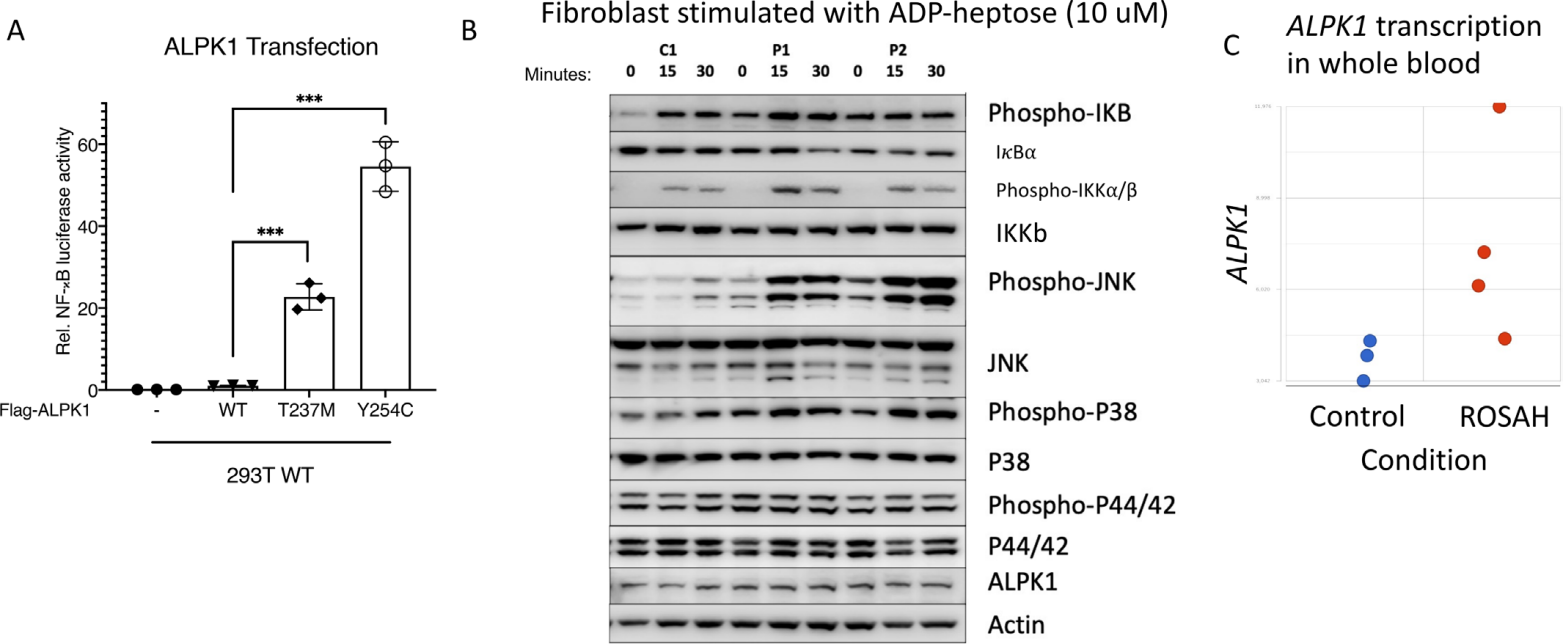
Gain-of-function mutations in *ALPK1* are associated with enhanced NF-κB activation in transfected cells and fibroblasts from patients with ROSAH syndrome. (A) 293 T cells were transiently cotransfected with an NF-B–responsive luciferase reporter gene and Flag-ALPK1 (wild-type or disease-associated mutant [T237M or Y254C]). Luciferase assay of NF-κB activation is shown as mean±SD. From three technical replicates (two-tailed unpaired Student’s t-test, ***p<0.001). (-) reflects transfection with empty vector. (B) Fibroblasts derived from patients with ROSAH syndrome were stimulated with ADP-heptose and whole cell lysates were immunoblotted against respective target proteins. Patient derived fibroblast showed increased levels of phospho-IκBα, increased degradation of IκBα, increased phospho-IKKα/β and increased MAPK activity (p38 and JNK). (C). Whole blood RNASeq data demonstrating ALPK1 mRNA expression was higher in untreated patients with ROSAH syndrome (red dots, n=4) as compared with controls (blue dots, n=3). ROSAH, retinal dystrophy, optic nerve oedema, splenomegaly, anhidrosis and headache.

We assessed the activity of mutant ALPK1 using an NF-κB luciferase assay. Transiently transfected mutant proteins had increased constitutive NF-κB activity relative to the wild-type protein. Additionally, the previously unreported Y254C variant showed a significantly higher NF-κB activity than the mutant T237M plasmid (figure 5A).

To explore the effect of the ALPK1 mutation ex vivo, we used T237M patient-derived fibroblasts from two unrelated patients to study the activity of the canonical NF-κB pathway in response to stimulation with the ALPK1 agonist ADP-heptose (the patient with the Y254C mutation declined skin biopsy). As compared with healthy controls, stimulated patients’ cells showed increased phosphorylation of IκBα, IKKα/β, and MAP kinases p38 and JNK, which are hallmarks of the activated canonical NF-κB pathway (figure 5B). Additionally, we observed higher mRNA expression of *ALPK1* and increased expression of NF-κB regulated genes in RNAseq data from patients with ROSAH syndrome (figure 5C and online supplemental figure 10).

### ROSAH syndrome mutations are associated with increased signal transducer and activator of transcription phosphorylation and expression of interferon-regulated genes

**Figure 6:**
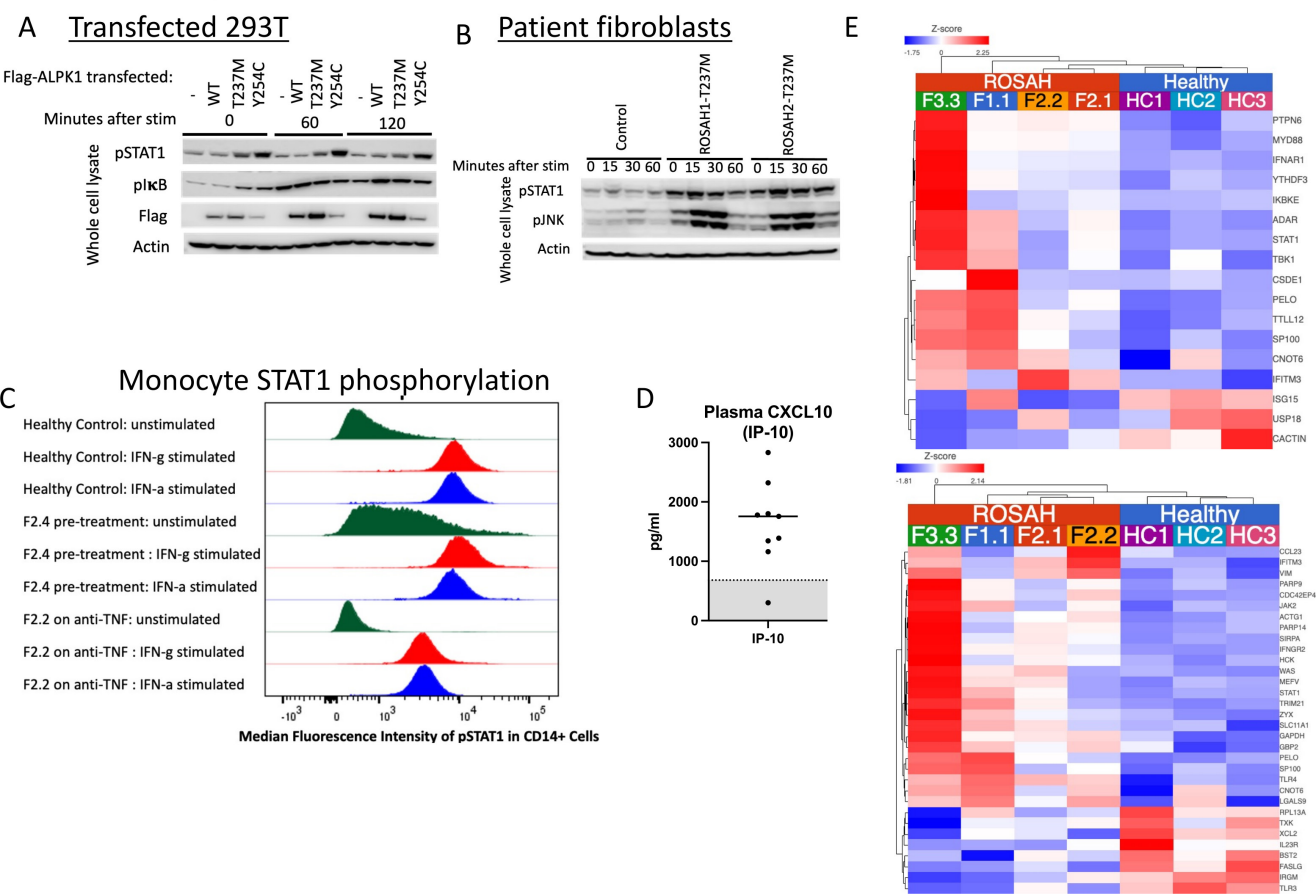
*ALPK1* mutations affect STAT1 phosphorylation, plasma levels of interferon-induced cytokines and transcription of interferon-regulated genes. (A, B) 293 T cells transiently transfected with ALPK1 variants (A) and ROSAH patient derived fibroblasts (B) were stimulated with ADP-heptose (5 uM) and whole cell lysates from both experiments were subjected to Western blotting for indicated proteins. Constitutive STAT1 phosphorylation (pSTAT) was observed in both transfected cells and patient fibroblasts. (-) reflects transfection with empty vector. (C) CD14-labelled monocytes from an untreated ROSAH patient (F2.4, middle of panel) showed constitutively phosphorylated STAT1 (pSTAT1) as compared with healthy control (top) and ROSAH patient treated with TNF-inhibitor (F2.2, bottom of panel). (D) Plasma CXCL10 (interferon-inducible protein 10 (IP-10)) as measured in patients F1.1, F2.2, F2.3, F2.4 and F7.1. Grey shaded area represents the mean plus or minus 2 SD from 114 healthy controls. (E) Heat map showing increased expression of interferon-regulated genes (type I: top (GO:0060337) and type II: bottom (GO:0034341)) in four untreated patients with ROSAH syndrome as compared with three healthy controls. Upregulated genes are shown in red and down-regulated genes in blue. ROSAH, retinal dystrophy, optic nerve oedema, splenomegaly, anhidrosis and headache; STAT1, signal transducer and activator of transcription.

Based on the presence of premature CNS basal ganglia mineralisation reminiscent of that seen in classic type-I interferonopathies including AGS, we were interested in assessing signal transducer and activator of transcription (STAT1) phosphorylation and expression of interferon-regulated genes.^21^ Immunoblotting for phospho-STAT1 in patient fibroblasts and in 293 T cells transfected with WT and mutant constructs revealed that ROSAH-associated mutations result in constitutive STAT1 activation (figure 6A, B). Unstimulated monocytes isolated from a pre-treatment ROSAH patient demonstrated increased STAT1 phosphorylation as compared with cells isolated from a healthy control and monocytes from a ROSAH patient treated with a TNF inhibitor (figure 6C).

We also observed elevated levels of CXCL10 in the peripheral whole blood samples (n=5 patients) and increased expression of interferon-regulated genes (n=4 patients) (figure 6D, E).

### Mouse model

Consistent with the observations in patients with ROSAH syndrome, knock-in mice with the *Alpk1* T237M mutation had elevated serum levels of CXCL1, CXCL10 and CCL2 (online supplemental figure 11a). At 16 weeks, mice did not have an increase in spleen size or weight (online supplemental figure 11b) and mice did not exhibit visual decline (online supplemental figure 11c, d) or evidence of retinal degeneration due to *Alpk1* mutation at up to 12 months of age (online supplemental figure 11e). However, we cannot exclude the possibility that retinal abnormalities could manifest in mice at a later age.

### Response to therapy

**Figure 7:**
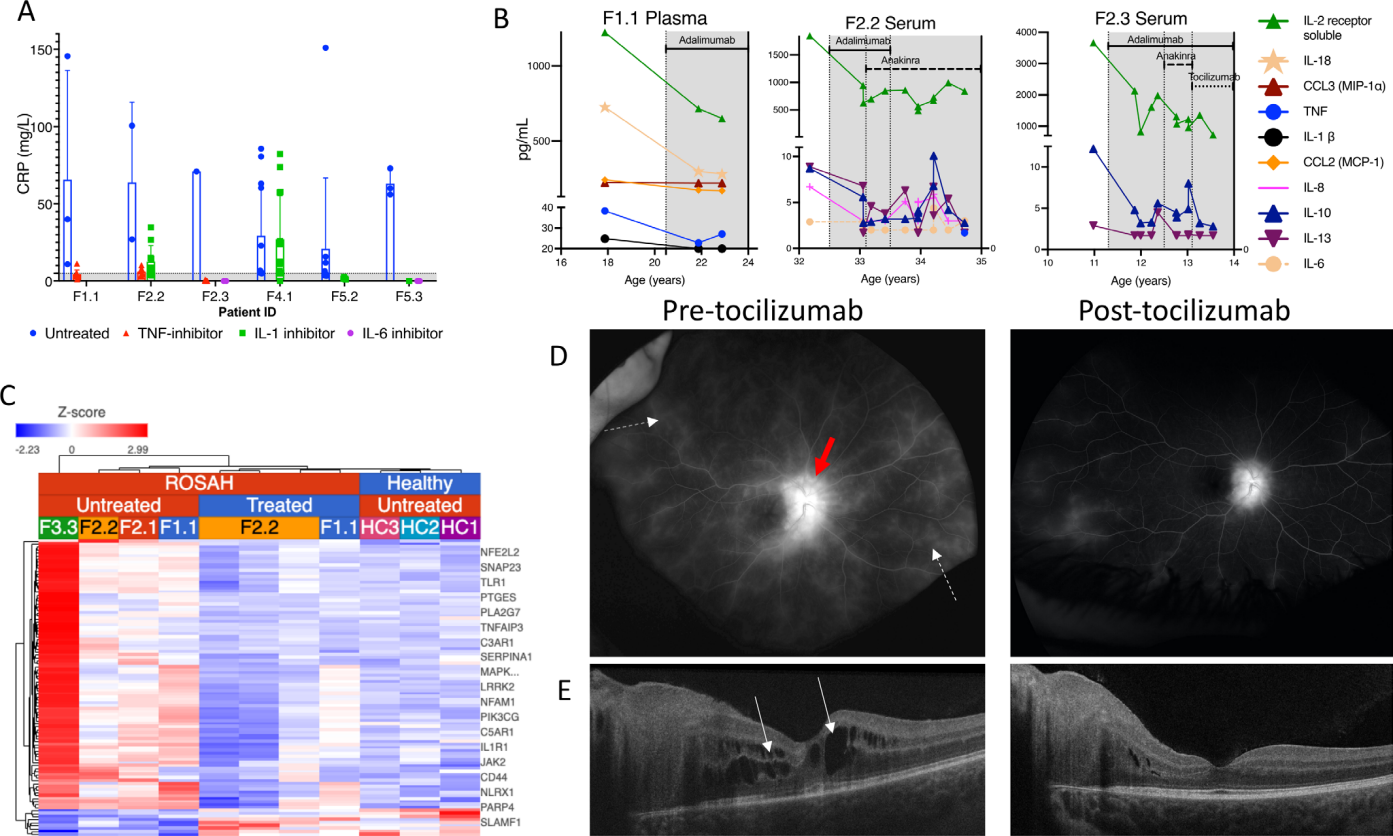
Response to anticytokine therapy. (A) Pretreatment and post-treatment CRPs for patients initiated on anticytokine therapy (n=6). Shaded zone represents normal range. (B) Pretreatment and post-treatment cytokines from patients F1.1 (plasma), F2.2 (serum), F2.3 (serum). Shaded area indicates time on anti-cytokine therapy. Specific therapies are as indicated in the figures. (C) Heatmap showing differentially expressed inflammatory response genes (GO: 0006954) in whole blood of pre-treatment (n=4) and post-adalimumab (n=2) patients with ROSAH syndrome. Patient F2.2 had post-treatment samples collected on three separate visits. Upregulated genes are shown in red, and downregulated genes in blue. Complete list of genes in online supplemental table 9. (D) Fluorescein angiography from patient F2.3 demonstrating retinal vasculitis (dotted white arrows) and disc leakage (solid red arrow) that improved after initiation of tocilizumab. (E) Optical coherence tomography from patient F2.3 demonstrating cystoid macular oedema (white arrows) that improved after initiation of tocilizumab. CRP, C reactive protein; ROSAH, retinal dystrophy, optic nerve oedema, splenomegaly, anhidrosis and headache.

Ten patients have been treated with anti-cytokine therapy (online supplemental table 1), and seven patients with systemic symptoms reported subjective improvement in at least one clinical feature of ROSAH syndrome. Patients F3.4 and F5.3 lacked subjective symptoms, and patient F13.1 denied subjective benefit but discontinued anti-IL-1 therapy (anakinra) after less than 1 week secondary to intolerable injection site reactions. Anti-TNF therapy (adalimumab) led to improvement in fatigue, headache, or arthralgia for four of four patients in whom these features were present. Additionally, three of these patients were noted to have normalised CRPs (figure 7A) and a decline in inflammatory cytokines (figure 7B) while on therapy. Six patients were treated with anti-IL-1 therapy (anakinra or canakinumab) and reported some improvement in subjective symptoms; however, serum CRP levels were not consistently suppressed.

Whole blood RNA sequencing was performed on paired pre- and post-treatment samples from patients F1.1 and F2.2. Prior to the initiation of treatment, patients with ROSAH syndrome demonstrated increased expression of many genes linked to inflammation (figure 7C, online supplemental table 8). After initiation of anti-TNF therapy, both patients had transcriptome changes consistent with decreased inflammation. Patients F2.1 and F3.3 are already blind and have declined treatment with anticytokine therapy.

Our ability to determine the impact of therapy on ocular disease was limited because most patients in this cohort already exhibited advanced retinal disease at the time of evaluation. However, two patients (F2.3 and F5.3) had substantially decreased intraocular inflammation after starting the IL-6 receptor antagonist, tocilizumab. Patient F2.3 had almost complete resolution of her cystoid macular oedema after 3 months of treatment with tocilizumab and this was maintained at 9 months on tocilizumab and fluorescein angiography showed significant improvement in retinal vascular leakage (figure 7D, E). After 5 months of tocilizumab treatment, patient F5.3 had decreased retinal vascular leakage and this improvement was maintained during 14 months of treatment. Patients F1.1 and F4.1 had continued visual decline with progressive constriction of visual fields despite adalimumab and canakinumab monotherapies, respectively. Subsequently, patient F4.1 was switched from canakinumab to sarilumab, the only subcutaneously administered IL-6 receptor antagonist locally available to the patient but within 1 week of the first 150 mg subcutaneous dose, developed grade 4 neutropenia (absolute neutrophil count <500/μL). Neutropenia persisted the following week and sarilumab was suspended (online supplemental figure 5a).

## Discussion

Although the initial report of ROSAH syndrome emphasised the visual manifestations associated with the disease, our work additionally establishes ROSAH syndrome as a disease of systemic inflammation caused by gain-of-function mutations in the innate immune receptor ALPK1. This conclusion is supported by both our in vitro work as well as our systematic analysis of inflammatory features in the largest cohort of patients reported to date. These findings have important implications for both basic science and clinical practice.

Our discovery of a second ROSAH-associated mutation occurring in the ligand-binding domain of ALPK1 emphasises the importance of this domain in protein activation and provides a solid foundation for establishing the pathogenicity of missense mutations affecting the region. While the exact impact of these mutations on protein structure and function remains to be elucidated, there is clearly a strong phenotypic overlap between patients with the recurrent p.T237M variant and the patient with the p.Y254C mutation. Additionally, our in vitro work demonstrates that both mutations are associated with increased innate immune activation as shown with enhanced NF-κB signalling and STAT1 phosphorylation in transfected cells and ADP-heptose stimulated patient fibroblasts.

The findings from this large, international cohort study provide valuable insights on the clinical spectrum of disease associated with mutations in *ALPK1* and highlight the possibility that patients with ROSAH syndrome may be currently unrecognised in cohorts of more common inflammatory disorders. We have found that ROSAH syndrome can present with periodic fevers, malaise, headaches, uveitis, deforming joint disease, abdominal pain, premature CNS mineralisation and focal meningeal enhancement on brain MRI and it has mimicked diseases including sJIA, sarcoidosis, neuro-Behçet’s disease, SjD and multiple sclerosis. We also found that untreated patients with ROSAH syndrome had frequent elevations of CRP and proinflammatory plasma cytokines including TNF and IL-6. Additionally, we have seen that diagnosis may be aided by the presence of ocular involvement, splenomegaly, decreased or inability to sweat or multiple dental caries, but none of these features is universal. While advanced retinal degeneration was common among adults in our cohort, three adults lacked significant visual impairment but suffered from other systemic inflammatory manifestations of the disease.

Patients’ clinical improvement on anticytokine therapies also highlights the role of immune activation in disease pathogenesis and emphasises the importance of referring patients with ROSAH syndrome for multidisciplinary evaluation and care. While all index patients in this cohort had routine ophthalmology care at the time of initial contact with the NIH, most patients did not have a regular provider experienced in management of systemic inflammation. Yet thorough examination revealed that many patients had elevations of serum CRP and non-ophthalmological indications to consider systemic immunomodulatory treatment including recurrent headaches, disabling episodes of fatigue, arthritis, abdominal pain, and AA amyloidosis. Most patients who received anti-TNF or anti-IL1 therapy reported subjective improvement in systemic symptoms. While these therapies may be appropriate for treating non-ocular inflammatory manifestation, there is no evidence that they are efficacious for treating intraocular inflammation or that they can influence progressive vision loss. Thus, additional prospective studies are needed to determine optimal treatment for this disease, but providers should consider alternative therapies in patients with active, vision-threating intraocular inflammation.

The IL-6 inhibitor tocilizumab has shown very promising results in patients F2.3 and F5.3. Both patients had intraocular inflammation that was unresponsive to TNF and IL-1 inhibition but showed dramatic improvement on tocilizumab, and we are actively seeking to determine if these results can be replicated in additional patients. It should also be noted that patients with ROSAH syndrome have an interferon gene expression signature, premature basal ganglia mineralisation and elevated CNS neopterin that suggest the disease may be an interferonopathy, which might indicate that patients would benefit from treatment with a JAK-inhibitor.^21 22^

Deep-phenotyping of this cohort illustrates the potential for monogenic diseases to advance our understanding on ALPK1’s role in human biology. Several clinical features of ROSAH syndrome, including short dental roots as well as the aberrant production of sweat, breast milk and saliva are not classically associated with inflammation but may reflect ALPK1’s role in ciliary functioning. Indeed, primary cilia are present in the dental epithelium and mesenchyme at various stages of tooth development, and the clinical and radiographic features of teeth in this cohort were very similar to past reports of dental anomalies in ciliopathy disorders.^23 24^ While, to date, no monogenic diseases have been categorised as both an autoinflammatory disease and a ciliopathy, this nosology may change as there are an increasing number of proteins that were initially labelled as ‘ciliary’ but have now also been observed at the innate immune synapse.^25 26^

The prevalence of clinical manifestations is this cohort was likely biased by the fact that all diagnostic genetic testing in the cohort was prompted by ophthalmological examination findings, and the prevalence of specific clinical features is likely to change as more patients without prominent ocular manifestations are screened for mutations in *ALPK1*. Additionally, the prevalence of disease features for deceased patients was limited to what could be recalled by their surviving children.

In conclusion, we have demonstrated that ROSAH syndrome is an autoinflammatory disease that can manifest with a spectrum of inflammatory features including recurrent fever, uveitis, deforming arthritis and cyclical cytopenias. For patients with advanced retinal degeneration, TNF inhibitors and IL-1 inhibitors can be considered for treatment of non-ocular disease manifestations including fevers, headaches, and arthritis. However, for patients with active intraocular inflammation, our findings indicate that tocilizumab may be the preferred treatment and future studies should be pursued to determine if this result is reproducible in additional patients. Continued study of ALPK1 function and ROSAH syndrome may also provide valuable insights for more common disorders of inflammation, such as gout or periodic fever, aphthous stomatitis, pharyngitis, cervical adenitis (PFAPA), where endogenous ligands may play a role as damage-associated molecular patterns.

## Data Availability

Data are available on reasonable request.
