# Supplementary material for: Gain-of-function mutations in ALPK1 cause an NF-κB-mediated autoinflammatory disease: functional assessment, clinical phenotyping and disease course of patients with ROSAH syndrome
Source: Ann Rheum Dis. 2022 Jul 22;81(10):1453–64. doi: 10.1136/annrheumdis-2022-222629 (PMC9484401; doi:10.1136/annrheumdis-2022-222629)
Supplement: Supplementary data [file annrheumdis-2022-222629supp001.pdf]

SUPPLEMENTAL MATERIAL

METHODS

Patient cohort and sample collection

Two patients were deceased. F3.1 died at age 40 from pancreatic cancer, and patient F5.1 died at age 59 from metastatic cancer of presumed colorectal source. Patient F2.4 has cerebral palsy and was unable to answer questions.

When feasible, serum, plasma, whole blood RNA, PBMC and fibroblasts were collected. Patients participated in recruiting other patients to the study and identification of outcome measures, and informed consent was obtained under an Institutional Review Board-approved protocol.

Identification of Y254C variant

Exome sequencing of genetically undiagnosed patients through the National Eye Institute clinical protocol (NCT02077894) were reviewed for novel candidate variants in ocular disease genes, including *ALPK1*, as previously described.(1) Sanger sequencing was used to confirm candidate variants.

CLINICAL PHENOTYPING

Eye exams

Eleven patients with ROSAH syndrome were examined at the National Eye Institute's (NEI) ophthalmology clinic including visual acuity measurements, anterior segment and dilated fundus exam. Patients who could cooperate with testing completed optical coherence tomography (OCT) (Cirrus HD-OCT; Carl Zeiss Meditec, Dublin, CA), color fundus imaging and fluorescein angiography (Optos ultrawide-field retinal imaging device; Dunfermline, Scotland).(2, 3)

Oral and dental exams

Seven patients with ROSAH syndrome were evaluated in the National Institute of Dental and Craniofacial Research (NIDCR) Dental Clinic using clinical and radiographic examination. Four patients underwent comprehensive oral and craniofacial examinations including unstimulated and stimulated salivary assessments and labial salivary gland (LSG) biopsies. Ultrasounds of the parotid and submandibular glands were performed using a GE Logic ultrasound machine using a 6-12 MHz linear transducer, and results were scored as previously described.(4, 5) Three patients had 3-dimensional (3D) intra oral scans (3shape A/S, Denmark) and cone beam computed tomography (CBCT) performed to further assess dental phenotype. Tooth width was measured on the intraoral scan, and this measurement was used to calibrate the scale in CBCT software. Tooth length was measured in the CBCT software (Planmeca Romexis, Planmeca USA Inc., IL, USA) in the axial orthogonal slices for individual teeth.

FUNCTIONAL STUDIES

Cytokine analysis

Analysis of cytokines, chemokines and other soluble biomarkers was performed on EDTA plasma, sera or cerebrospinal fluid (CSF).

CRP was measured in the clinical laboratories of local health care facilities. Plasma cytokines (tumor necrosis factor (TNF)-alpha, interleukin (IL)-6, interferon (IFN)-beta, IL-10, monocyte

chemoattractant protein-1, IL-1 beta, IFN-gamma, macrophage inflammatory protein-1 alpha, granulocyte-monocyte colony stimulating factor, IL-2 receptor alpha soluble, IFN-alpha, IL-18) were measured through Mayo Clinic Laboratories. Serum and CSF cytokines (TNF-alpha, IL-2, IL-2 receptor soluble, IL-12, IFN-gamma, IL-4, IL-5, IL-10, IL-13, IL-17, IL-1 beta, IL-6, IL-8) were measured through ARUP Laboratories. Additionally, an extended panel of serum and plasma cytokines were tested in-house. Samples were diluted 1:3 in universal assay buffer and analyzed with the ProcartaPlex Cytokine & Chemokine 34-Plex Human Panel 1A according to the manufacturer's instructions.

Functional analysis of human neutrophils and monocytes
Internalization of microbes by neutrophils and monocytes was performed using pHrodo-labeled *Staphylococcus aureus* as previously described.(6)

Whole blood RNA sequencing for gene expression analysis
Total RNA was extracted from whole blood collected in PAXgene Blood RNA tubes using the PAXgene Blood RNA Kit (PreAnalytiX) per manufacturer's instructions. RNA quality was assessed using the Agilent TapeStation and quantified by the Nano Drop-1000 Spectrophotometer.

A total of 1000 ng of RNA (RNA integrity number [RIN] ≥7) per sample was used for cDNA library preparation using the NEBNext Ultra II Directional RNA library preparation kit with NEBNext Poly(A) mRNA magnetic isolation module and NEBNext Globin and rRNA Depletion kit (E7765, E7490 and E7755, New England Biolab). Sequencing was performed on an Illumina NovaSeq6000 System in a 2 x 150 bp paired-end mode. Sequenced reads were mapped to the human reference genome (GRCh38) using Hisat2.

BAM files were imported to Partek Flow (www.partek.com) and quantified to hg38. All count data were normalized using DESeq2. Differentially expressed genes (DEGs) were selected as those with a false discovery rate <0.05, and heat maps showing gene expression patterns were generated using Partek. Ingenuity Pathway Analysis (IPA) software (Qiagen) was used to perform pathway enrichment and network analysis. The RNA-sequencing data are uploaded and available online (Gene Expression Omnibus: GSE198443).

Cell cultures and plasmids
293T cells (negative for mycoplasma, originally obtained from the American Type Culture Collection) and skin fibroblast derived from patients with ROSAH syndrome and healthy donors were grown in Dulbecco's modified Eagle's medium (Life Technologies) plus 10% fetal bovine serum and 1x antibiotics (Life Technologies).

The wild-type (WT) ALPK1 plasmid was generated as previously described and the T237M and Y254C mutants were generated by site-directed mutagenesis and validated by means of Sanger sequencing.(7)

NF-κB luciferase reporter assay
Using Lipofectamine 2000 according to the manufacturer's instructions (Invitrogen), HEK293T cells (seeded at 35,000 cells per well in 96-well plates) we co-transfected with a NanoLuc®

reporter under a NF-κB-responsive element, a firefly expression vector, and an ALPK1 cDNA construct carrying either a WT or mutant sequence. Twenty-four hours after transfection, luciferase activity was measured using the Nano-Glo® Luciferase Assay System (Promega), and the NanoLuc® luciferase activity was normalized against the firefly luciferase activity for control of transfection efficiency and cell number. The reporter activity was then normalized to the result of WT construct.

Cell stimulation and immunoblotting

ADP-heptose (Invivogen) was used to stimulate fibroblast and transfected 293T cells (5-10 uM) for indicated times. Whole cell lysates were prepared using ice-cold 1x cell lysis buffer (Cell Signaling) supplemented with complete protease inhibitors. Immunoblotting was conducted using specific antibodies as described previously. ImageJ was used to analyze the immunoblotting images.

Antibodies specific for Phospho-IKKα/β (#2697), IKKα (#11930), IκBα (#4814, #9242), Phospho-IκBα (#2859), Phospho-p38 MAPK (#4511), Phospho-p44/42 MAPK (Erk1/2) (#4370), Phospho-SAPK/JNK (#4668), p44/42 MAPK (Erk1/2) (#4695), SAPK/JNK (#9252), p38 MAPK (#8690), HRP-linked anti-rabbit IgG (#7074), HRP-linked anti-mouse IgG (#7076), Phospho-Stat1 (Tyr701) (#D4A7), Rabbit mAb (#7649); DYKDDDDK Tag (D6W5B) rabbit mAb (#14793) were obtained from Cell Signaling, ALPK1 (PA5-55616) was obtained from Invitrogen and Actin (sc-1615), Anti-OctA-Probe Antibody (sc-166355) were obtained from Santa Cruz.

***In vitro* pSTAT1 phosphorylation assay**

PBMCs from ROSAH patients and healthy donors were isolated by Ficoll (Ficoll-Paque PLUS; GE Healthcare) density-gradient centrifugation. STAT1 phosphorylations was assessed by treating PBMCs with IFN-α or IFN-γ (Cell Signaling Technology, Boston, MA) (200ng/ml) at 37°C for 20 minutes. The stimulation was stopped by addition of 1X Lyse/Fix Buffer (BD Biosciences, San Diego, CA). The PBMCs were then washed with PBS and permeabilized in ice-cold Phosflow Perm Buffer III (BD Biosciences, Sand Diego, CA) for 30 minutes in the dark. Cells were washed with FACS buffer (PBS, pH 7.4, 1% BSA, 4 mM EDTA, 0.2% NaN_3). Monoclonal antibodies to CD3, CD4, CD8, and CD14 (BD Biosciences, San Diego, CA) were used to identify $CD4^+$ T cells, $CD8^+$ T cells and monocytes respectively. STAT1 phosphorylations were evaluated with 20 mL of Alexa Fluor647 pSTAT1 antibody (BD Biosciences, San Diego, CA). Flow cytometric analysis was performed on a FACS Fortessa (BD Immunocytometry, San Jose, CA) with Diva software. Data were analyzed using FlowJo software (Treestar, Ashland, OR). Median fluorescence intensity (MFI) of the corresponding pSTAT1 were calculated for each cell subset.

Mice

All animal studies were performed in accordance with guidelines from Ministry of Health, China or the National Institutes of Health and were approved by the Institutional Animal Care and Use Committee of National Institute of Biological Sciences, Beijing or National Human Genome Research Institute, Bethesda, MD or National Eye Institute, Bethesda, MD, respectively. To generate *Alpk1*$^{T237M/ T237M}$ mice, gRNA (ACAGGGCATTTCCACATCAC) targeting exon 9 of *Alpk1* and donor

atgagagaattccagtctgatttgtttgtttttcctgacagggcatttccATGAGTTTAggcatactggcagacatctttgtttccatgag caaaaccgattatgaaaa were used. *In vitro*-transcribed guide RNA, Cas9 mRNA and donor were co-microinjected into C57BL/6N-derived zygotes. The tail genomic DNA of each offspring was amplified with the forward primer 5'-AGCCCAACTTCAAAGTAGCC -3' and the reverse primer 5'- CATCGAGAGAGGCTGGGATA -3'. Sanger sequencing was performed to analyze the PCR products and identify the founders with T237M mutation. Founders with the same mutation were intercrossed to obtain homozygous *Alpk1*$^{\mathrm{T237M}}$ mice.

Because C57BL/6N mice harbor an *rd8* mutation in the *Crb1* gene that results in a form of retinal degeneration, the C57BL/6N *Alpk1*$^{\mathrm{T237M}}$ mice were crossed with C57BL/6J mice that lacked the *rd8* mutation.(8) Eight *Alpk1*$^{\mathrm{T237M/T237M}}$ *Crb1*$^{\mathrm{rd8/rd8}}$ mice underwent ophthalmologic evaluation with fundus imaging and histologic evaluation of eyes from sacrificed mice. Three of the mice were imaged and sacrificed at 10 months of age and five of the mice were imaged and sacrificed at 7 months of age.

Five *Alpk1*$^{\mathrm{T237M/WT}}$ *Crb1*$^{\mathrm{rd8/WT}}$ mice were evaluated by ERG at 9 and 12 months of age. At 12 months of age, the mice were also evaluated for retinal degeneration by fundus imaging and optical coherence tomography (OCT) and Optodrum was used to assess visual acuity, as previously published.(9, 10)

Four *Alpk1*$^{\mathrm{T237M/WT}}$ *Crb1*$^{\mathrm{WT/WT}}$ mice were evaluated by ERG at 6 and 9 months of age. At 9 months of age, the mice were also evaluated for retinal degeneration by fundus imaging and OCT and Optodrum was used to assess visual acuity. Four wild-type control mice were evaluated by ERG at 6 months of age, but one died at 8 months of age so only 3 control mice were evaluated at 9 months of age.

Statistical analysis

Continuous variables are presented as means with standard deviations or medians with interquartile ranges and were compared with the use of parametric tests as appropriate.

BMJ Publishing Group Limited (BMJ) disclaims all liability and responsibility arising from any reliance placed on this supplemental material which has been supplied by the author(s)

SUPPLEMENTAL FIGURES AND TABLES

Supplemental Table 1: Demographic and treatment information of patients with ROSAH syndrome.

Supplemental Table 1: Characteristics of patients with ROSAH syndrome

ID	Current age (years)	Sex	Nationality	Ophthalmologic diagnosis	Previous treatment	Current treatment
F1.1	23	Male	United States	optic nerve elevation, retinal degeneration, uveitis	Colchicine, IVIG	Adalimumab, Methotrexate
F2.1	61	Female	United States	optic nerve elevation, retinal degeneration	None	None
F2.2	34	Female	United States	optic nerve elevation, retinal degeneration, uveitis	Adalimumab	Anakinra, Budesonide slurries Omeprazole
F2.3	13	Female	United States	optic nerve elevation, uveitis	Anakinra	Adalimumab, Tocilizumab
F2.4	8	Female	United States	Pale optic discs, retinal scars and pre-retinal peripheral membranes in patient with retinopathy of prematurity status post-laser and vitrectomy*	None	Levetiracetam, Oxcarbazepine, Clonazepam
F3.1	Died at 40-pancreatic ca.	Male	United States	optic nerve elevation, retinal degeneration		
F3.2	47	Male	United States	optic nerve elevation, uveitis		Colchicine, allopurinol
F3.3	45	Male	United States	optic nerve elevation, retinal degeneration, uveitis	Chlorambucil, methotrexate, cyclosporin, mycophenolate mofetil, acetazolamide, oral prednisone	None
F3.4	14	Male	United States	optic nerve elevation, uveitis	acetazolamide, oral prednisone	Adalimumab
F3.5	7	Male	United States	optic nerve elevation		None
F4.1	26	Male	Italy	optic nerve elevation, retinal degeneration, uveitis	Colchicine, oral steroids, canakinumab, sarilumab	Anakinra
F5.1	Died at 59-metastatic ca. of presumed colorectal source	Male	France	**		
F5.2	42	Male	France	optic nerve elevation, uveitis	Oral steroids	Anakinra
F5.3	14	Male	France	optic nerve elevation, uveitis, retinal detachment	Anakinra, Adalimumab	Tocilizumab

F6.1	62	Female	Brazil	optic nerve elevation, retinal degeneration, uveitis	Oral prednisone	None
F6.2	28	Female	Brazil	optic nerve elevation, retinal degeneration, uveitis	None	None
F7.1	60	Male	United States	optic nerve elevation, retinal degeneration, uveitis	Oral prednisone	Metformin
F8.1	38	Male	The Netherlands	optic nerve elevation, retinal degeneration	steroids, cyclosporine, octreotide, acetazolamide, mycophenolate mofetil, ocular triamcinolone injections, infliximab, adalimumab	None
F9.1	36	Male	United States	optic nerve elevation, uveitis	None	Lutein
F9.2	9	Female	United States	optic nerve elevation	None	None
F9.3	6	Male	United States	optic nerve elevation	None	None
F10.1	42	Female	England	optic nerve elevation, retinal degeneration	Oral prednisone	None
F11.1	51	Female	Norway	optic nerve elevation		None
F11.2	27	Male	Norway	**		None
F11.3	25	Female	Norway	optic nerve elevation, retinal degeneration, uveitis	Oral prednisone	Colchicine
F12.1	43	Female	Japan	optic nerve elevation, retinal degeneration, uveitis, retinal detachment	Adalimumab, oral steroids	Colchicine, NSAIDs
F13.1	43	Female	United States	optic nerve elevation, retinal degeneration, uveitis	methotrexate, mycophenolate mofetil, anakinra (discontinued after 1 week secondary to intolerable site reaction)	Oral prednisone

*Ocular exam limited by patient’s ability to cooperate.
**Ocular exam not performed

Supplemental Figure 1: **Pedigrees of the 13 families with mutations in *ALPK1* leading to ROSAH syndrome**.

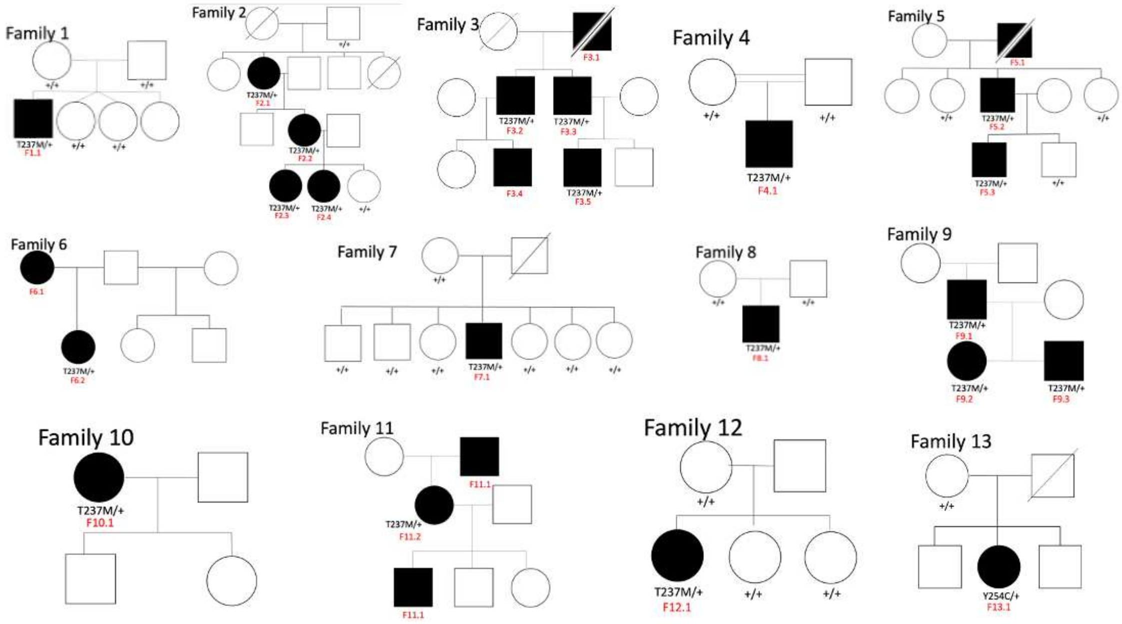

Pedigrees of 27 patient from 13 families described in this cohort. Clinically affected individuals are shaded, and results are presented for those examined by Sanger sequencing. Variant status is indicated below each individual for whom sequencing data was obtained (T237M indicates allele coding for [p.Thr237Met) and Y254C indicates allele coding for [p.Tyr254Cys]; + indicates normal allele). A diagonal line indicates a deceased individual.

Supplemental Figure 2: **Dot plot depicting age of onset for patients with functional visual deficits**.

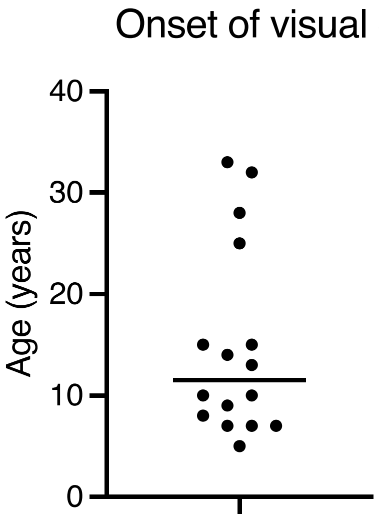

Supplemental Table 2: Post-splenectomy pathology reports for patients F2.2, F5.2, F7.1, F11.2 F11.3 and F12.1.

ID	Age (years)	Spleen size at resection	Splenic pathology
F2.2	13	1,320 grams; 26 x 15 x 6 cm	Normal architecture with expanded red pulp and mild histiocytic hyperplasia. Scattered megakaryocytes but no other evidence of extramedullary hematopoiesis (EMH). (IHC stains: CD79a, CD3, CD163, CD68, MPO, CD4, CD8; Congo red negative)
F5.2	21	600 grams; 18x10.5x4.5 cm	Hyperplastic aspect of white pulp. Discretely congested red pulp.
F7.1	59	1,599 grams, 27 x 17 x 8 cm	Red pulp congestion
F11.2	21	528 grams, 18x10.5x5 cm	Atrophic white pulp. Congestion, some hyperemia and expanded red pulp.
F11.3	20	688 grams, 16x6x10 cm	Thickened capsule, thickened walls with sclerosis in walls. Red pulp is expanded, dilated sinusoids and veins. Hemosiderin containing macrophages. Severe congestion.
F12.1	28	3100 grams	Red pulp is highly congested and white pulp is mildly atrophic

Normal spleen weight for adult male is 28-226 grams and adult female is <230 grams.(11, 12)

Supplemental Figure 3: **MRI abnormalities in ROSAH syndrome.**

A White matter changes

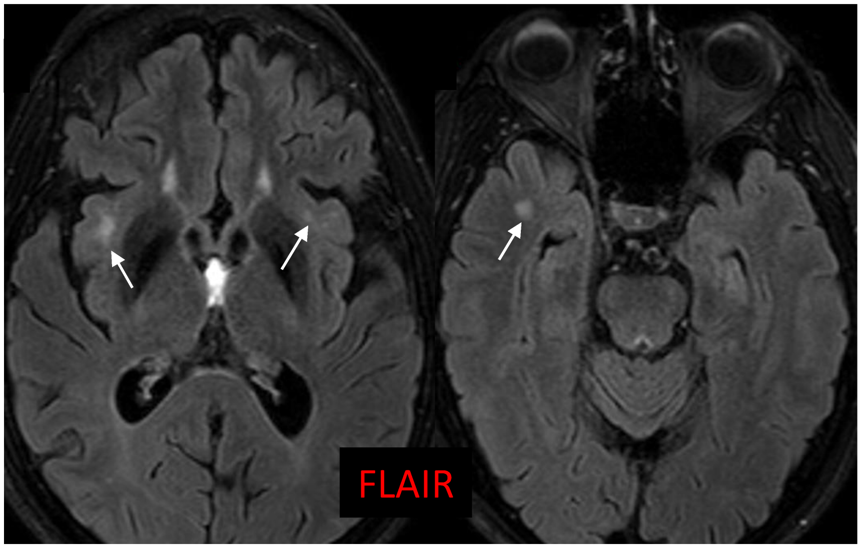

F7.1

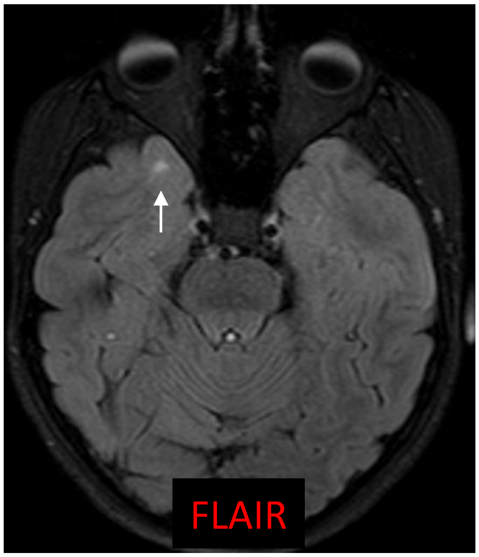

F2.3

A, cont.

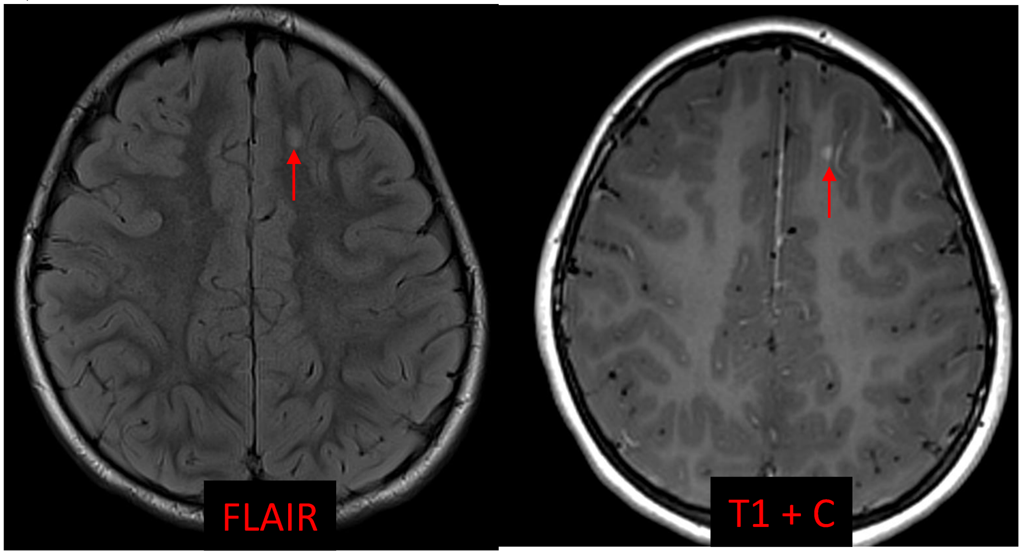

F3.4

A. Bilateral focal hyperintensities (small white arrows) on fluid-attenuated inversion recovery (FLAIR) images in the subinsular regions and right temporal lobe (small white arrows) of patient F7.1. Focal FLAIR hyperintensity in the subcortical white matter of the right temporal lobe of patient F2.3. Focal area of increased FLAIR signal with associated enhancement (red arrows) is seen in the left frontal subcortical white matter in patient F3.4.

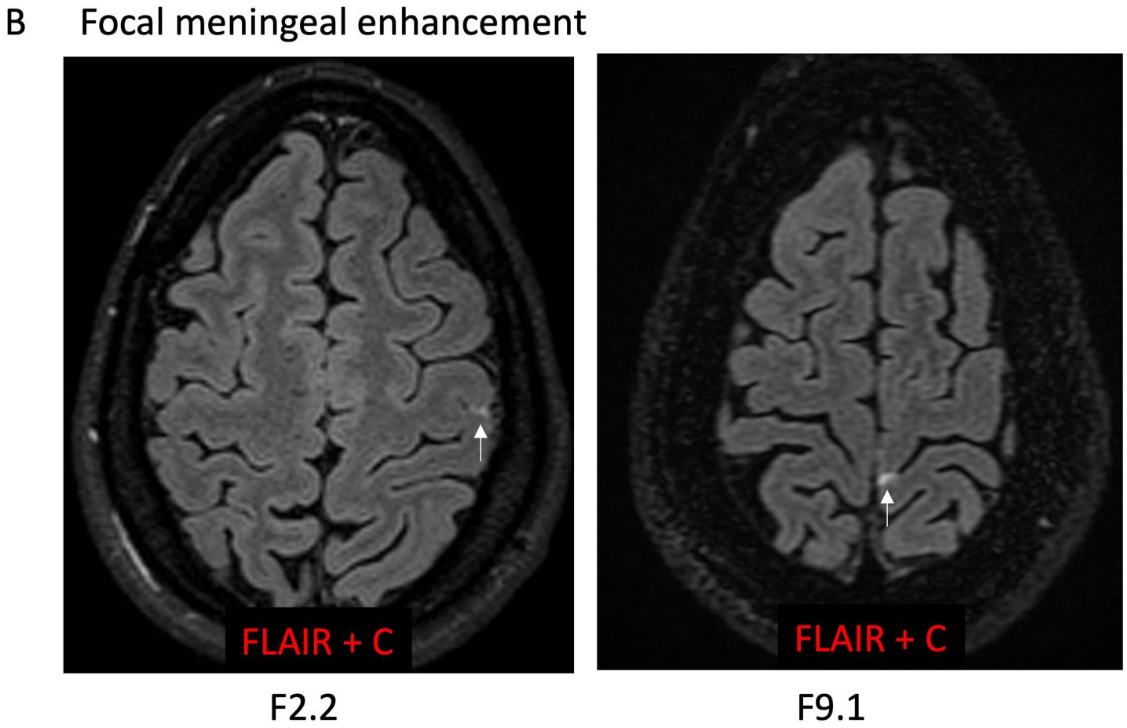

B. Focal meningeal enhancement (white arrows) on post-contrast FLAIR imaging for patients F2.2 (left) and F9.1 (right).

C

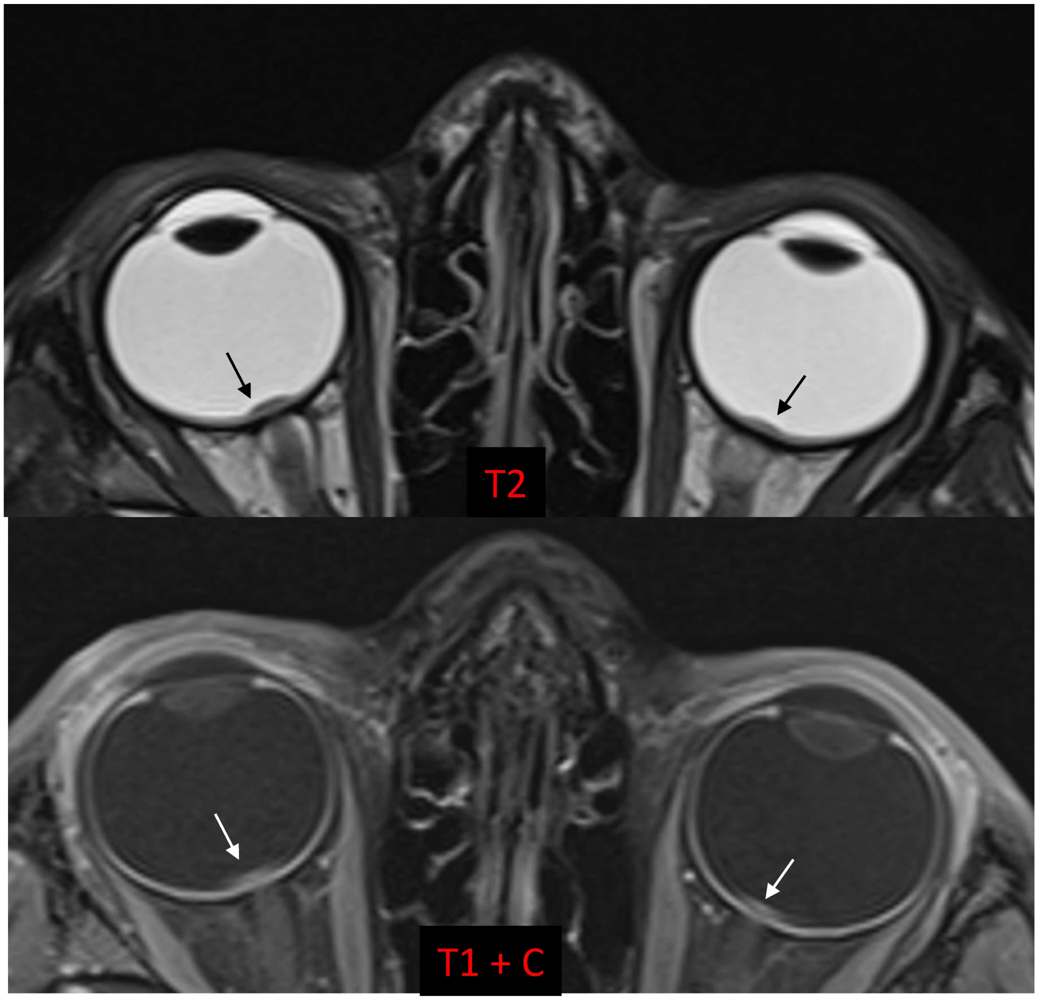

F3.4

C, cont.

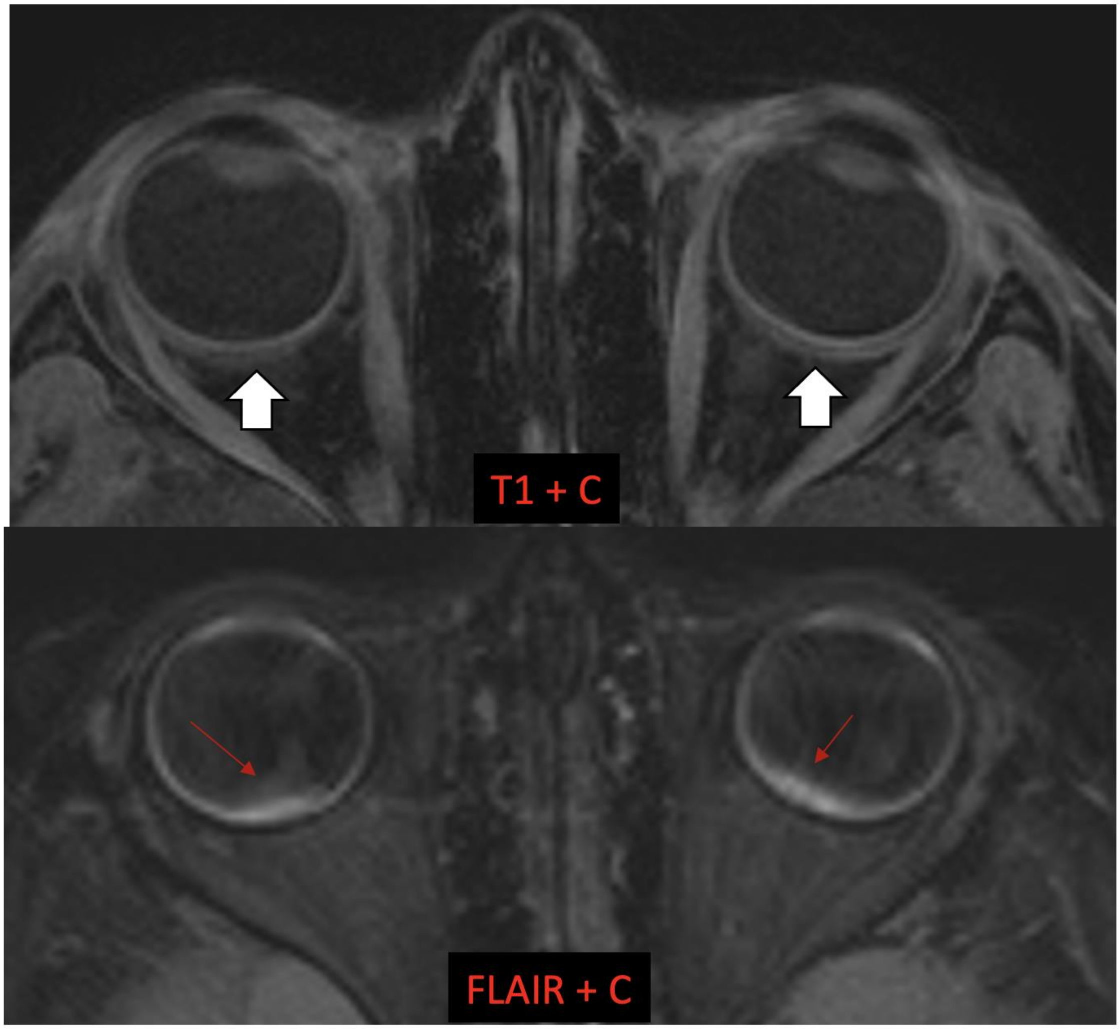

F2.3

C, cont.

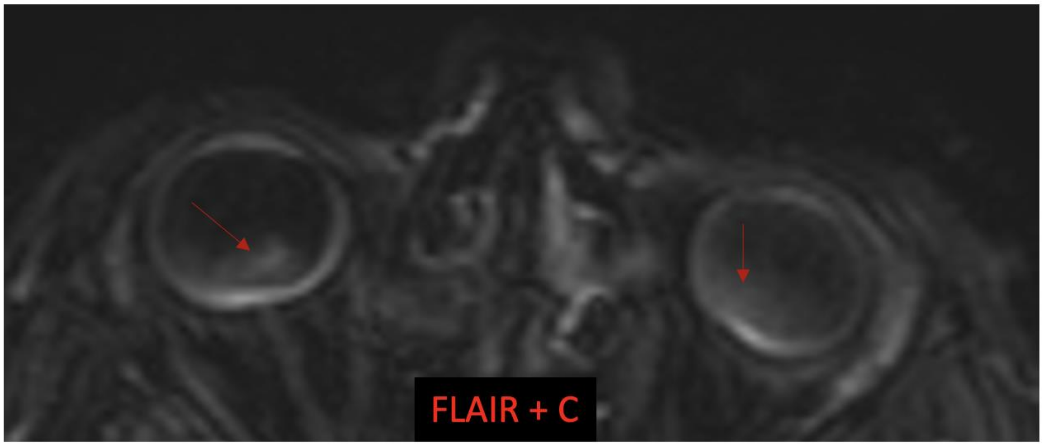

F4.1

C. MRI of the orbits showed papilledema on T2 weighted (small black arrows) and post contrast T1-weighted images (small white arrows) of patient F3.4. Axial post contrast T1 and FLAIR images show thickening/enhancement of the posterior aspects of the globes (thick white arrows) for patient F2.3. Leakage of contrast material into the vitreous humor on delayed post contrast FLAIR images in patients F2.3 and F4.1(red arrows).

Supplemental Figure 4**: Oral pathology in ROSAH.**

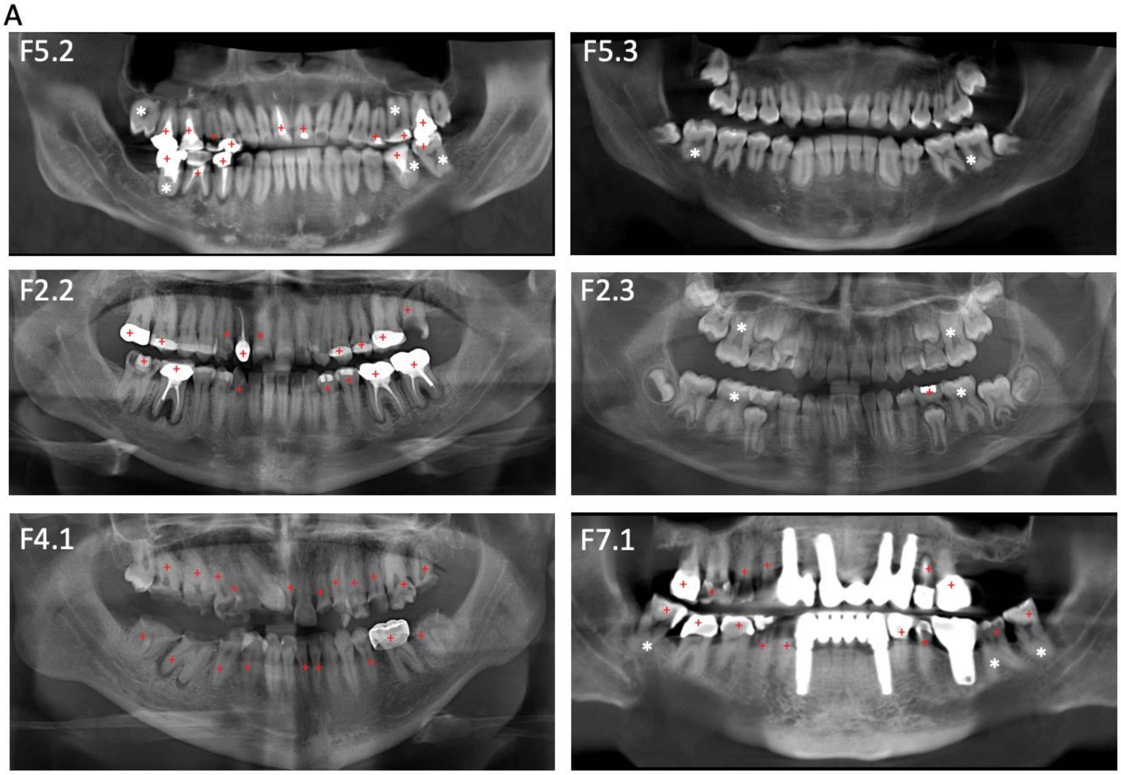

A. Panel of dental panoramic X-rays from ROSAH individuals. Short roots are noted in all except F2.2. Multiple dental restorations present in F2.2 and F5.2, possibly due to enamel defects or caries. Multiple dental implants in F7.1. *marks teeth with taurodontism. +marks decayed or restored teeth.

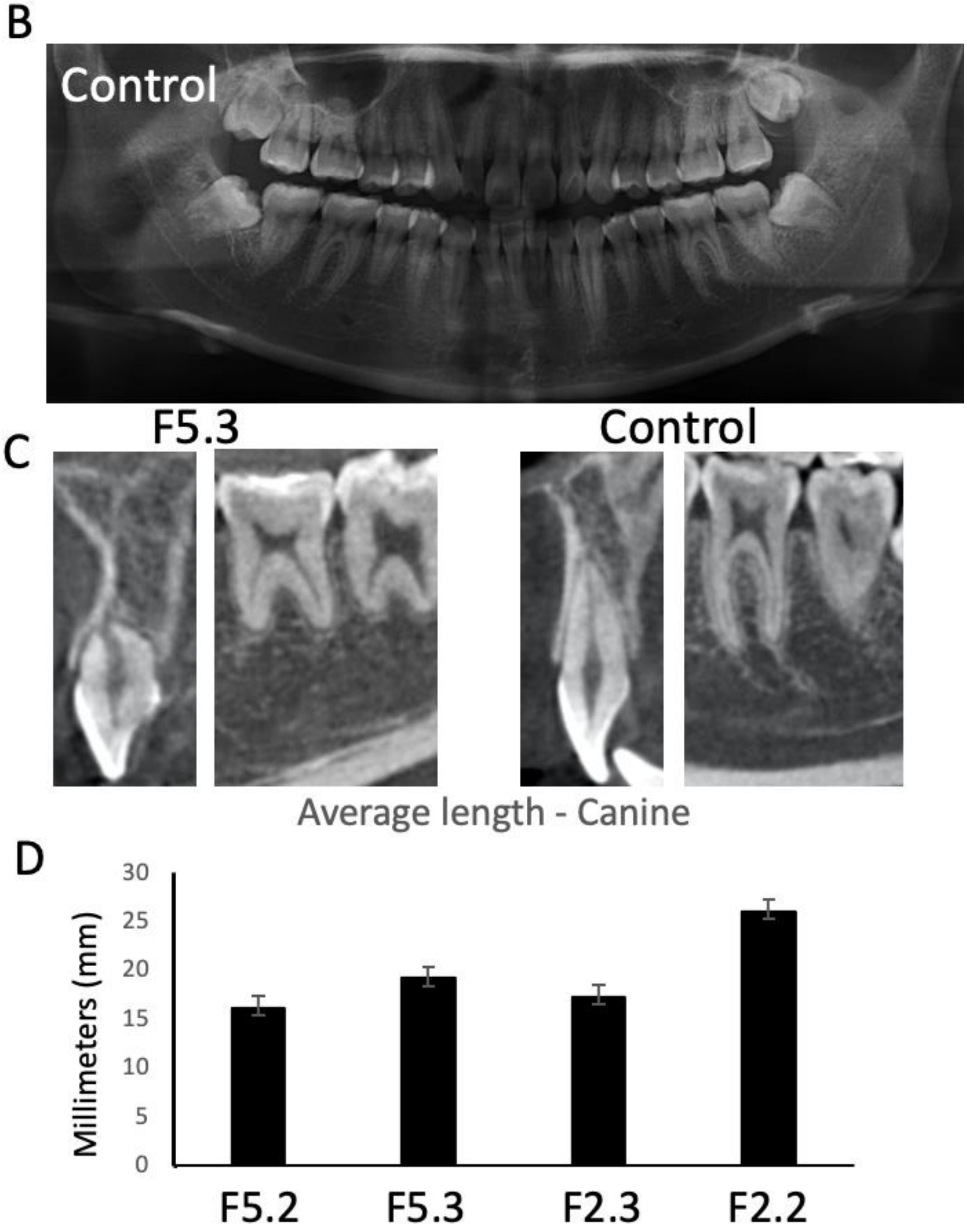

B. Panoramic X-rays from 14-year-old healthy male for comparison as control.
C. 2D orthogonal slices of maxillary central incisors and mandibular molars in F5.3 (left) and healthy control (right) comparing abnormal teeth roots.
D. Quantification of canine length in four individuals showing the variation in length.

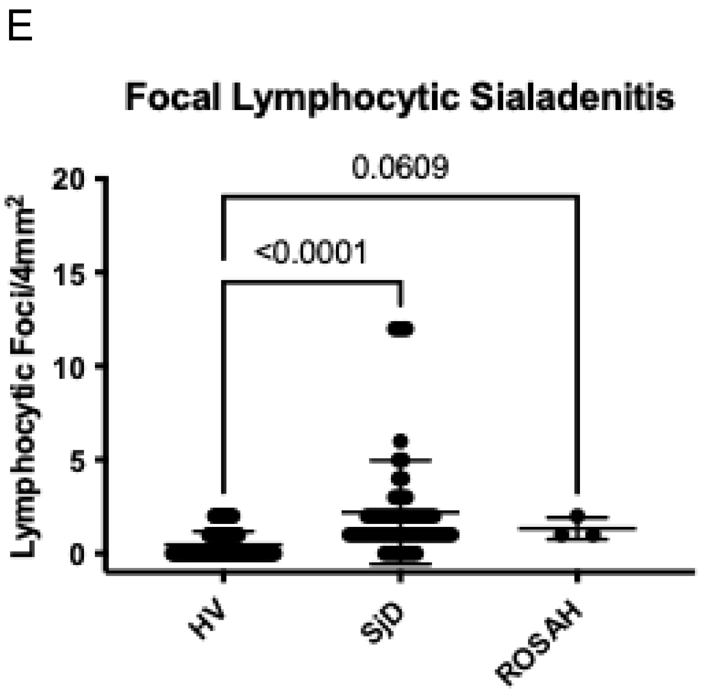

E. Focal lymphocytic sialadenitis (FLS) scores of salivary gland histopathology from healthy volunteers (HV), Sjögren’s Disease (SjD), and patients with ROSAH syndrome.

Supplemental Table 3

American College of Rheumatology-European League Against Rheumatism (ACR-EULAR) Classification Criteria for Sjögren's Disease

Classification criteria (positive range)	Points for positive score	Patient 5.2 (points)	Patient 5.3 (points)	Patient 2.2 (points)	Patient 7.1 (points)
Focus score (≥ 1 foci/mm^2)	3	1(3)	N/A*	1(3)	2(3)
Anti-SSA (Ro) (positive)	3	Negative (0)	Negative (0)	Negative (0)	Negative (0)
Unstimulated whole saliva (≤ 1.5 ml/15 min)	1	3.4218 (0)	1.9443 (0)	0.7644 (1)	4.4442 (0)
Ocular staining score (≥ 5)	1	5 (1)	3 (0)	N/A	4 (0)
Schirmer test (≤ 5 mm/5 min)	1	11 (0)	18 (0)	N/A	5 (1)
Total score (≥ 4 meet the criteria for SjD)		4	0	4	4

*biopsy not performed on Patient 5.3, a minor.

Supplemental Figure 5: **Inflammation and cytopenias in ROSAH syndrome.**

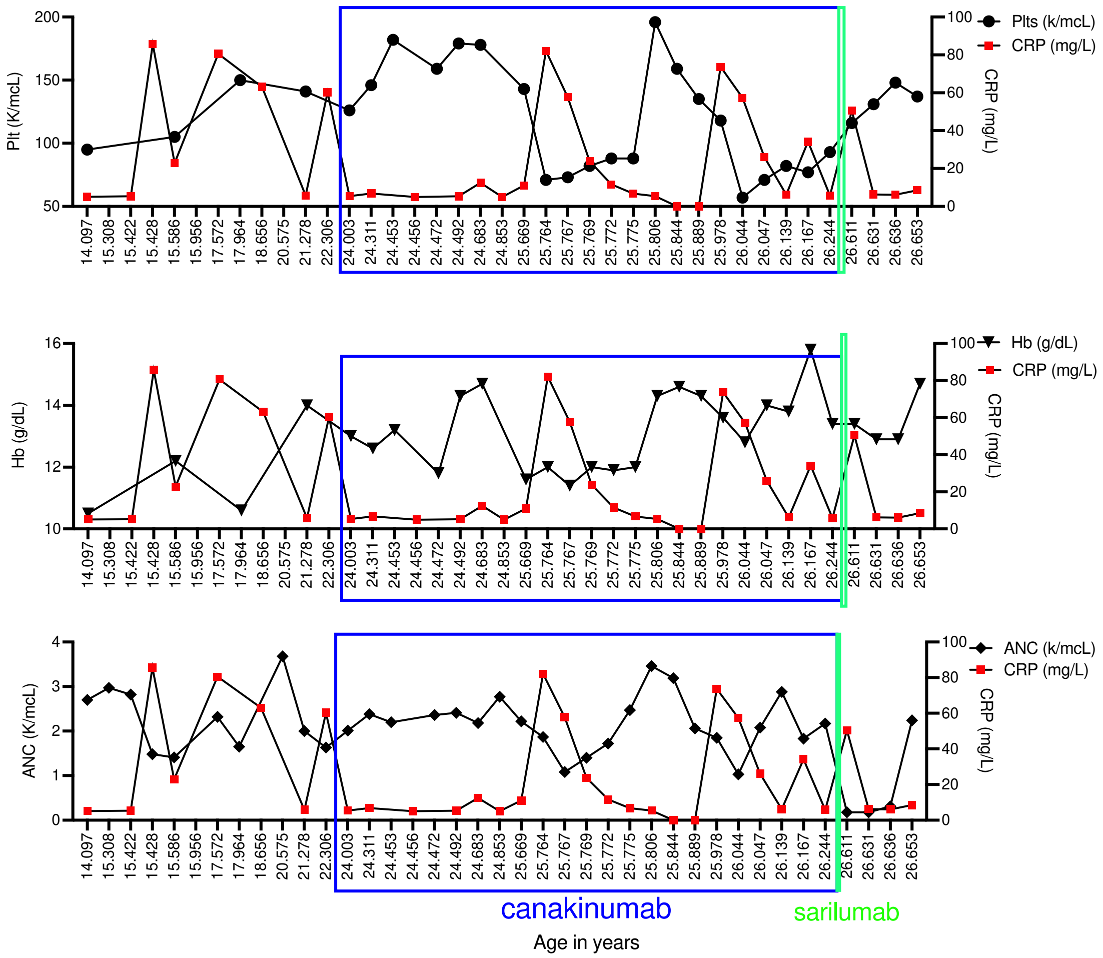

A). Cell counts and serum CRP levels for patient F4.1 demonstrate that his cytopenia frequently coincide with CRP elevations. Cell counts are plotted on level axis and shown in black and serum CRP levels are plotted on right axis and shown in red. Blue boxes outline time patient was taking canakinumab. Green line denotes administration of single dose of sarilumab that was suspended after development of neutropenia.

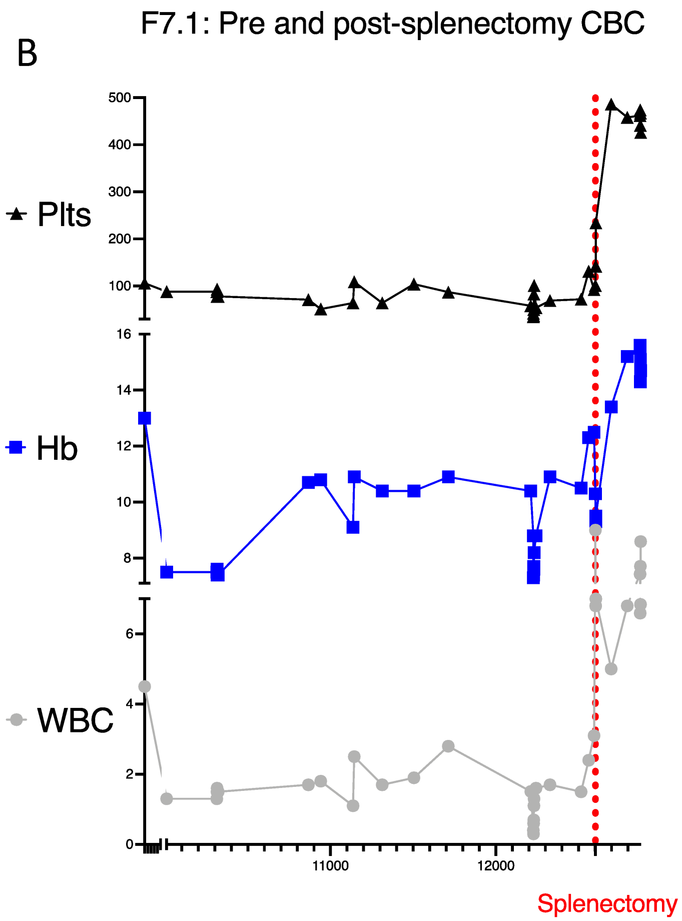

B. Post-splenectomy, cytopenias appear to resolve but immune activation continues.
B) Pre- and post-splenectomy blood counts for patient F7.1. The timing of splenectomy is indicated by the vertical red dotted line.

Supplemental Figure 6:

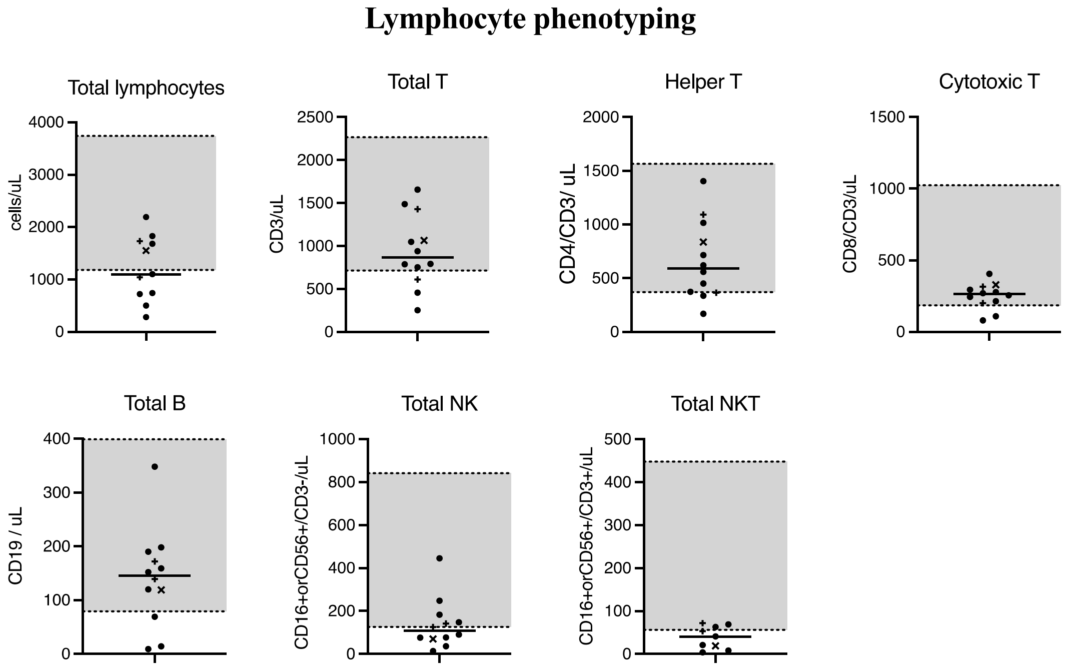

Immunophenotyping of blood cells in patients with ROSAH syndrome (n=12) is depicted. Normal reference ranges are indicated by the gray shaded areas. The indicated reference ranges do not apply to all plotted values. Three pediatric patients are plotted and indicated by the symbols as followed: “X” Indicates value from 8-year-old (Patient F2.4). “+” Indicates values from patient between 11-18 years old (Patients F2.3 and F5.3). For pediatric patients, the following reference ranges can be used: For 6-10 years old - Total lymphocyte count 1500-7000/uL, total T 700-4200/uL, Helper T 300-2000/uL, Cytotoxic T 300-1800/uL, Total B 200-1600/uL, NK 90-900/uL. For 11-18 years old -Total lymphocyte count 1000-5200/uL, total T 800-3500/uL, Helper T 400-2100/uL, Cytotoxic T 200-1200/uL, Total B 200-600/uL, NK 70-1200/uL. Also, patient F8.1 had an NK cell count of 90/uL and the normal reference range for NK cell count from the laboratory performing the test was reported to be 90-600/uL.

Supplemental Figure 7: **ALPK1 transcription in immune cells and pHrodo dye-based detection of phagocytosis**.

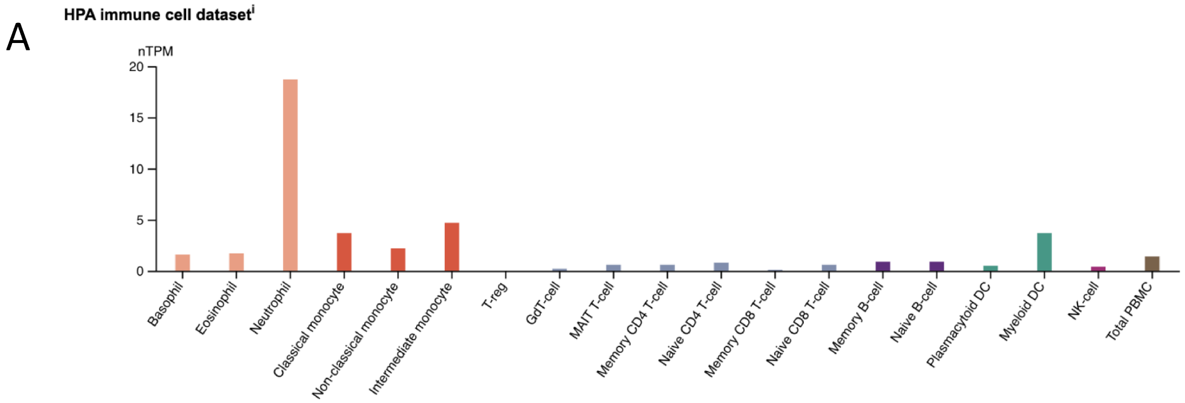

A. ALPK1 expression in human immune cells. (13)

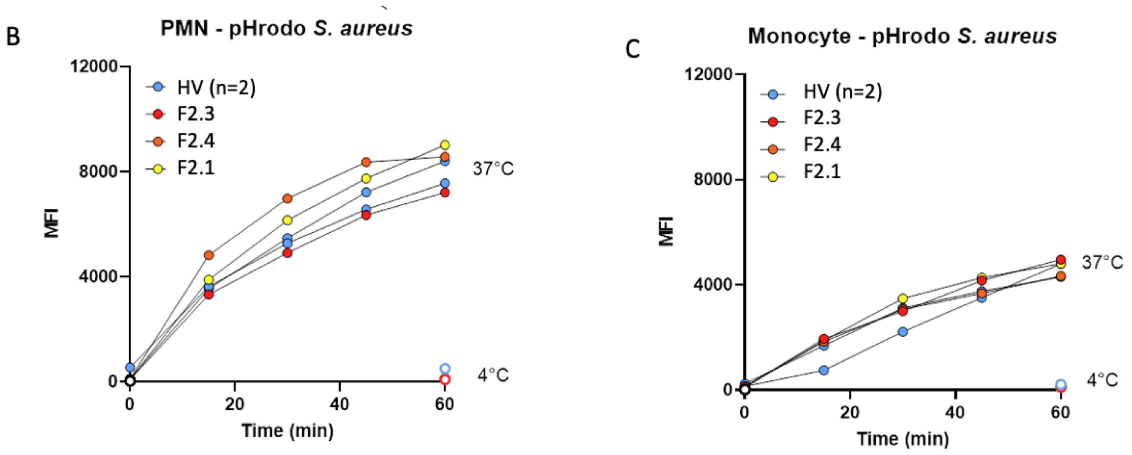

B-C. Ingestion of *Staphylococcus aureus* labelled with pH-sensitive dye by polymorphonuclear leukocytes (PMNs) (b) and monocytes (c) was assessed by flow cytometry. MFI: mean fluorescence intensity. HV: healthy volunteer.

Supplemental Figure 8: **Serum immunoglobulins**

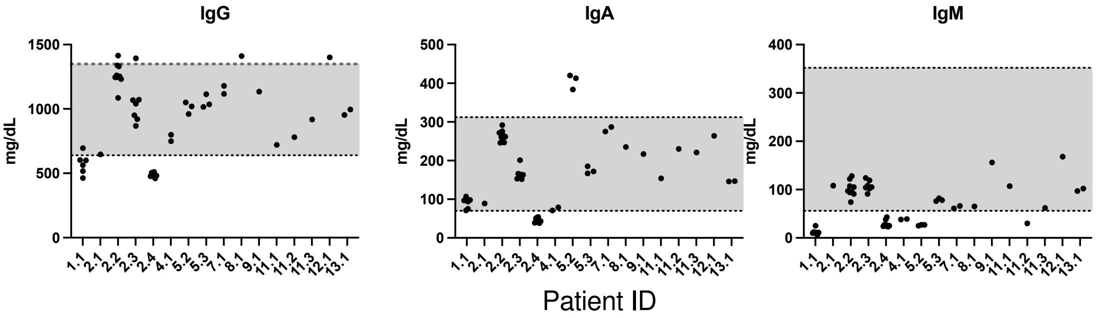

Serum immunoglobulins measured in 16 patients with ROSAH syndrome. As compared to age-adjusted normal values, patient F1.1 and F2.4 had low IgG and patients F1.1, F2.4, F4.1, F5.2 and F11.2 had low IgM (14). Gray shaded areas represent the indicated normal reference range for individuals >10 years of age. *denotes pediatric patient. Normal reference range for patient between age 6-8 years is IgG: 633-1280 mg/dL, IgA: 45-236 mg/dL, IgM: 52-242 mg/dL.

Supplemental Table 4: **Autoantibodies in ROSAH syndrome cohort**. Most patients with ROSAH syndrome lacked high-titer autoantibodies.

	ANA	RF	anti-CCP	anti-ds DNA	ENA screen (anti-RNP, SM, SSA, SSB, Jo-1, Scl-70)	Anti-SS-A (Ro)	Anti-SS-B (La)
F1.1	negative	negative	negative	negative	negative		
F2.1	negative	negative		negative	negative		
F2.2	1:320 Homogeneous	negative	negative	negative	negative		
F2.3	1.4 EU (positive)	negative	negative	negative	negative		
F2.4	negative	negative	negative				
F3.3	Non-reactive x 3	negative					
F3.4	Non-reactive x 3	negative					
F4.1	<0.1	negative	negative	negative	negative		
F5.2	<1:80	negative	negative	negative	negative		
F5.3	1:320 Homogeneous	negative	negative	negative	negative		
F6.2	negative	negative	negative	negative	negative		
F7.1	negative	negative	negative	negative	negative	negative	negative
F8.1	negative	negative		negative	negative	negative	negative
F9.1	negative	negative	negative			negative	negative
11.1	negative	negative	negative				
11.2	negative	negative	negative				
11.3	negative	negative	negative				
12.1	<40 (negative)	negative	negative	negative			negative
F13.1	0.2 (negative)	negative	negative				

Supplemental Figure 9: **Inflammatory signature in patients with ROSAH syndrome.**
A

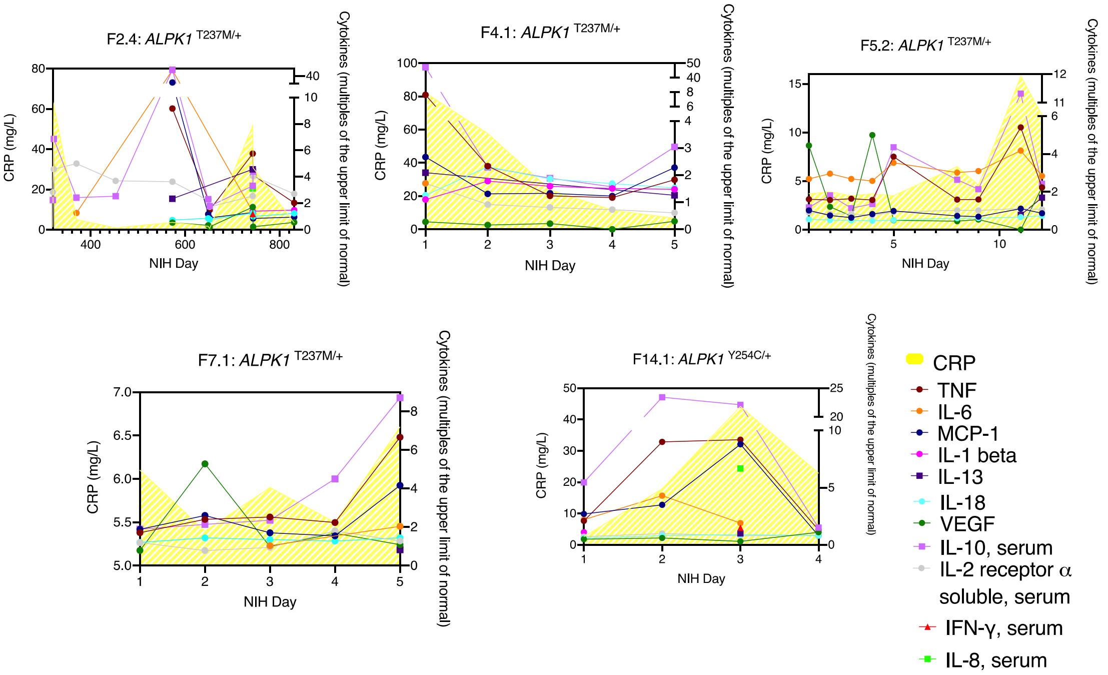

A) Serum CRP as well as plasma and serum cytokines and chemokine levels were trended in 3 untreated patients (Patient F2.4, F5.2, F7.1), one patient with continued fever and CRP elevations despite anti-IL-1 therapy (patient F4.1) and one patient on 5 mg of prednisone daily (patient F13.1). CRP (mg/dL) is shown in yellow and plotted on the left y axis. Cytokines and chemokines are graphed as multiples of the upper limit of normal (ULN). ULN determined by reference laboratory performing the assay.

B

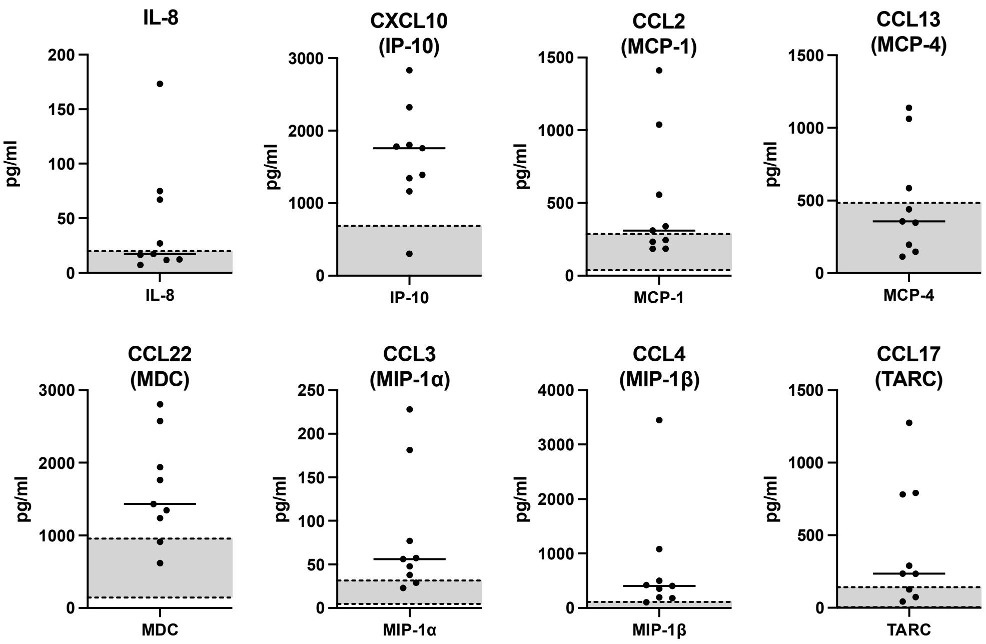

B) Additional plasma cytokines and chemokines measure in patients F1.1, F2.2, F2.3, F2.4 and F7.1. Gray shaded areas represent the mean plus or minus 2 standard deviation from 114 healthy controls.

C

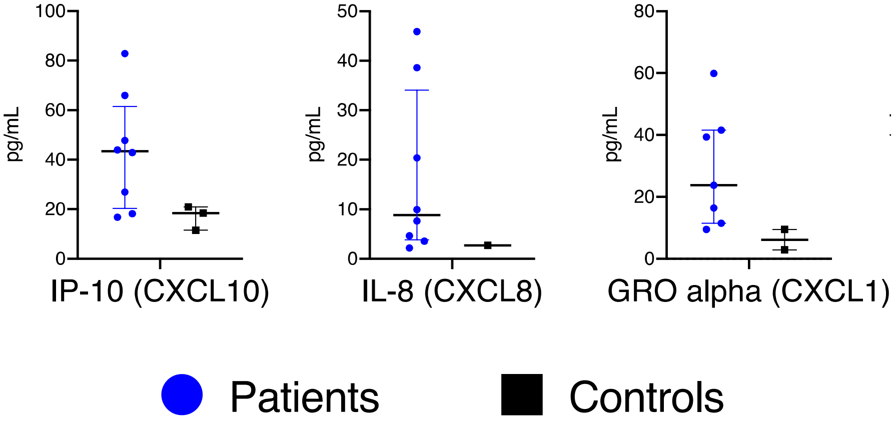

C) Additional serum cytokines and chemokines measure in patients F2.1, F2.2, F2.3, F2.4, F3.3 and F3.4. Patients with ROSAH syndrome are plotted on left with blue circles. Health controls are plotted on right with black squares (n=3).

Supplemental Table 5:

	Reference range	F1.1	F2.4
Interferon-gamma Positive CD4	8-24%	21	7 (L)
Interferon-gamma Positive CD8	20-48%	23	16 (L)
Interferon-gamma Positive NK	48-80%	56	75
Interferon-gamma Positive NKT	18-64%	40	Insufficient NKT cells in sample
TNF-alpha Positive CD4	39-67%	66	49
TNF-alpha Positive CD8	17-61%	28	24
TNF-alpha Positive NK	29-61%	39	55
TNF-alpha Positive NKT	23-73%	49	Insufficient NKT cells in sample
IL-4 Positive CD4	0-3.7%	0.8	0.6
IL-4 Positive CD8	0-3%	0.5	0.0
IL-4 Positive NK	<1.6%	0.4	0.0
IL-4 Positive NKT	0-3.1%	0.4	Insufficient NKT cells in sample

Stimulated ROSAH leukocytes did not display increased intracellular cytokines (n=2). Whole blood cells from patient F1.1 and F2.4 were incubated with Phorbol 12-Myristate 13 Acetate and Ionomycin for 4 hours at 37°C to stimulate cytokine production. Activation was carried out in the presence of Brefeldin A, to inhibit intracellular transport, causing all cytokines produced during the activation to be retained inside the cell. The activated cells were stained with surface monoclonal antibodies for phenotyping, fixed, permeabilized and stained with antibodies to the cytokines IFN-γ, TNF-α and IL-4. The cells were then analyzed by flow cytometry. Percent positive results for IFN-γ, TNF-α and IL-4 were reported for CD4, CD8, NK and NKT cells. At the time of cell collection, patient F1.1 was on adalimumab and patient F2.4 was not receiving any anti-cytokine treatment. (L) indicates value below reference range.

BMJ Publishing Group Limited (BMJ) disclaims all liability and responsibility arising from any reliance placed on this supplemental material which has been supplied by the author(s)

Supplemental Table 6: **Selected studies of CSF obtained by lumbar puncture from patients with ROSAH syndrome.**

	**F2.2 (33 yo)**	F3.4 (10 yo)	F10.1 (33 yo)
	Results [normal reference]		
Opening pressure (cm H_2O)	17 [6-25, adult]	26.6 [12-28, pediatric]	22 [6-25, adult]
WBC (cells/mm^3)	3 [0-5]	2 [0-7]	4 (75% lymph, 23% mono, 2% unclassified; Large mononuclear cells with basophilic cytoplasm and fine chromatin)
Protein (mg/dL)	39 [23-38]	23 [25-45]	60 [5-55]
CSF neopterin (nmol/L)	71 [8-28]	65 [7-40]	Not measured
Current treatment	TNF-inhibitor	None	None
Cytology			Numerous medium-sized cells with round to folded and indented nuclei, prominent nucleoli and moderately abundant basophilic cytoplasm, some with cytoplasmic vacuoles and granules, consistent with activated monocytes and lymphocytes. The overall findings are consistent with a reactive process.

Supplemental Table 7: **Paired serum and CSF cytokines for patient F2.2**.

	CSF		Serum	
	Result (pg/mL)	Reference range	Result	Reference range
CRP			7.3	0-4.99 mg/L
TNF- α	<1.7	<=1.7	7	<=7.2
IL- 2	<2.1	<=2.1	<2.1	<=2.1
IL- 2 receptor α, soluble	36.3	<=26.8	627.6	175.3-858.2
IL- 12	<1.9	<=1.9	<1.9	<=1.9
IFN- γ	<4.2	<=4.2	<4.2	<=4.2
IL- 4	<2.2	<=5.2	<2.2	<=2.2
IL- 5	<2.1	<=2.1	<2.1	<=2.1
IL-10	5.7	<=12.7	<2.8	<=2.8
IL-13	38.3	<=7.3	<1.7	<=2.3
IL-17	<1.4	<=4.6	<1.4	<=1.4
IL-1 β	<6.5	<=6.5	<6.5	<=6.7
IL- 6	7.1	<=7.5	<2	<=2.0
IL- 8	186.3	4.6-283.5	<3.0	<=3.0

Supplemental Figure 10: **NF-κB signature**.

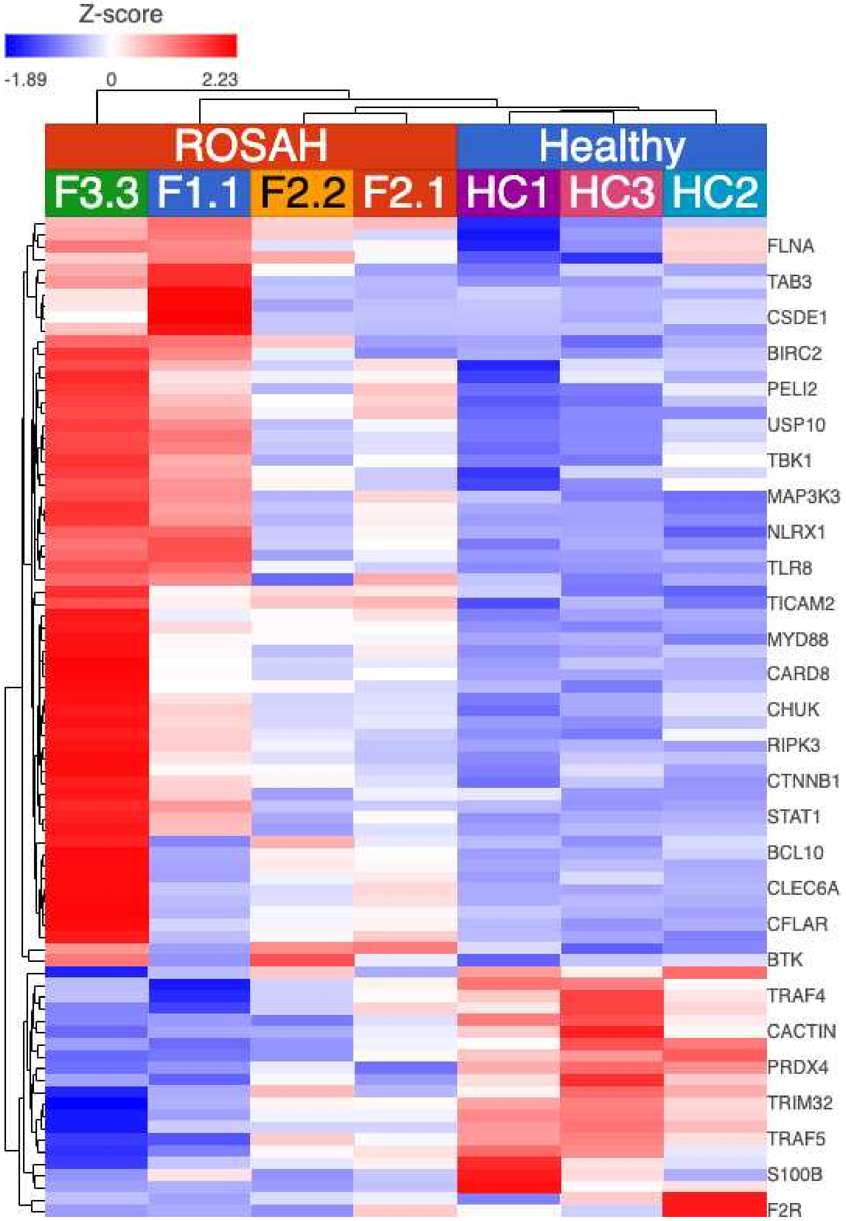

Heatmap showing differentially expressed NF-κB response genes (GO:0007249) in whole blood of pre-treatment (n=4) patients with ROSAH syndrome and healthy volunteers (n=3). Up-regulated genes are shown in red, and down-regulated genes in blue. For patients F1.1 and F3.3, samples were collected on days for which sera and plasma cytokines were also significantly elevated, consistent with disease flare.

Supplemental Figure 11: **Mice harboring ROSAH-associated mutations in *Alpk1* demonstrated serum cytokine and chemokine elevations similar to those seen in patients with ROSAH syndrome, but splenomegaly, visual deficits, and retinal degeneration were not observed**.

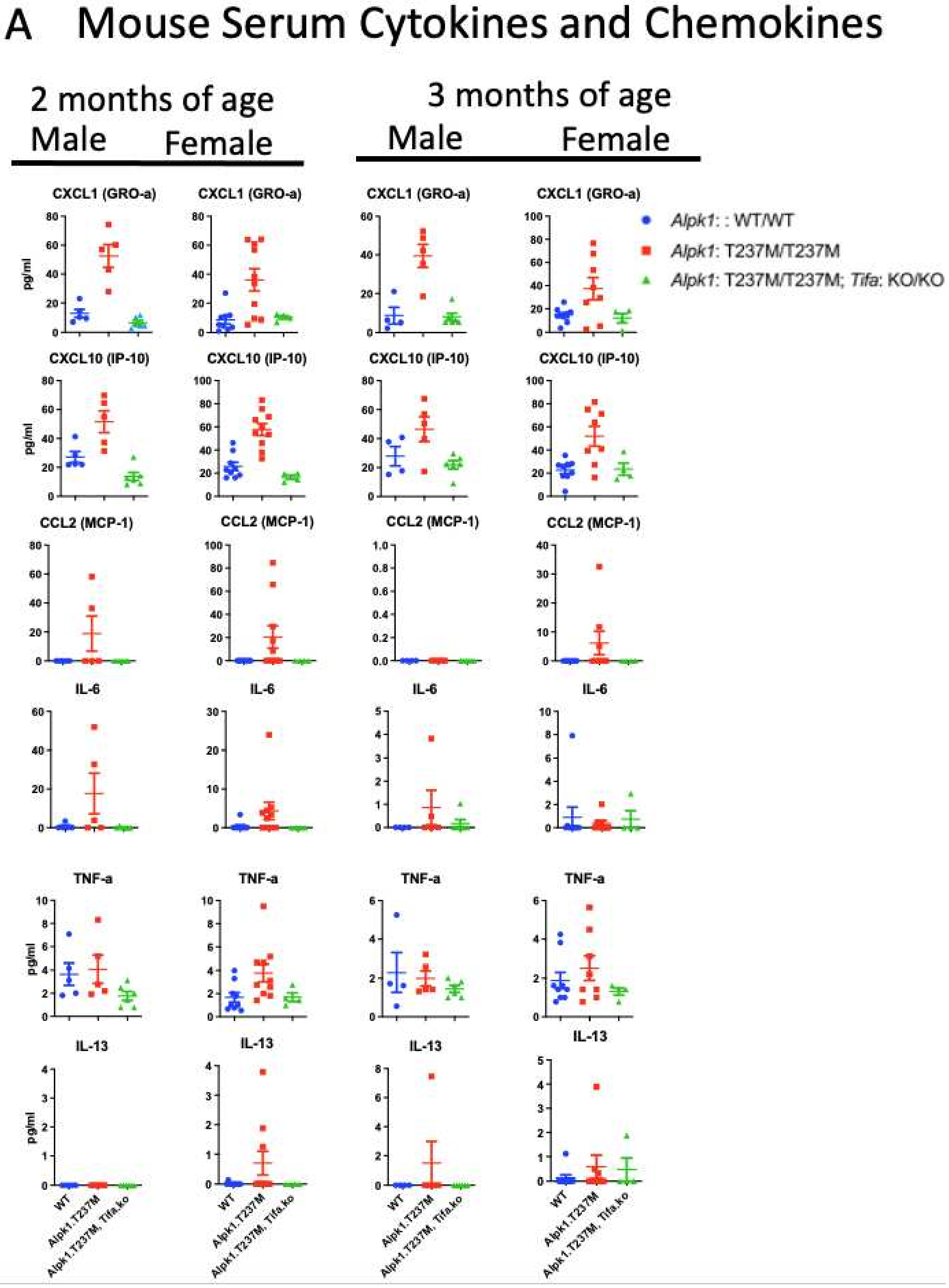

Activation of NF-κB by ADP-Heptose involves ALPK1 and is reported to be TIFA (TRAF-interacting protein with forkhead-associated domain) dependent (7).

A. Cytokine profiling by multiplex immunoassay was performed on serum from wild-type (WT) C57BL/6 mice, *Alpk1*$^{T237M/T237M}$ mice and *Alpk1*$^{T237M/T237M}$/*TIFA*$^{-/-}$ mice at 2 and 3 months of age. Cytokine concentrations in the serum shown as mean ± s.e.m. As compared to WT and *Alpk1*$^{T237M/T237M}$/*TIFA*$^{-/-}$ mice, *Alpk1*$^{T237M/T237M}$ mice showed elevation of inflammatory biomarkers including CXCL1, CXCL10 and CCL2, suggesting that this inflammation is secondary to activation of the ALPK1-TIFA axis.

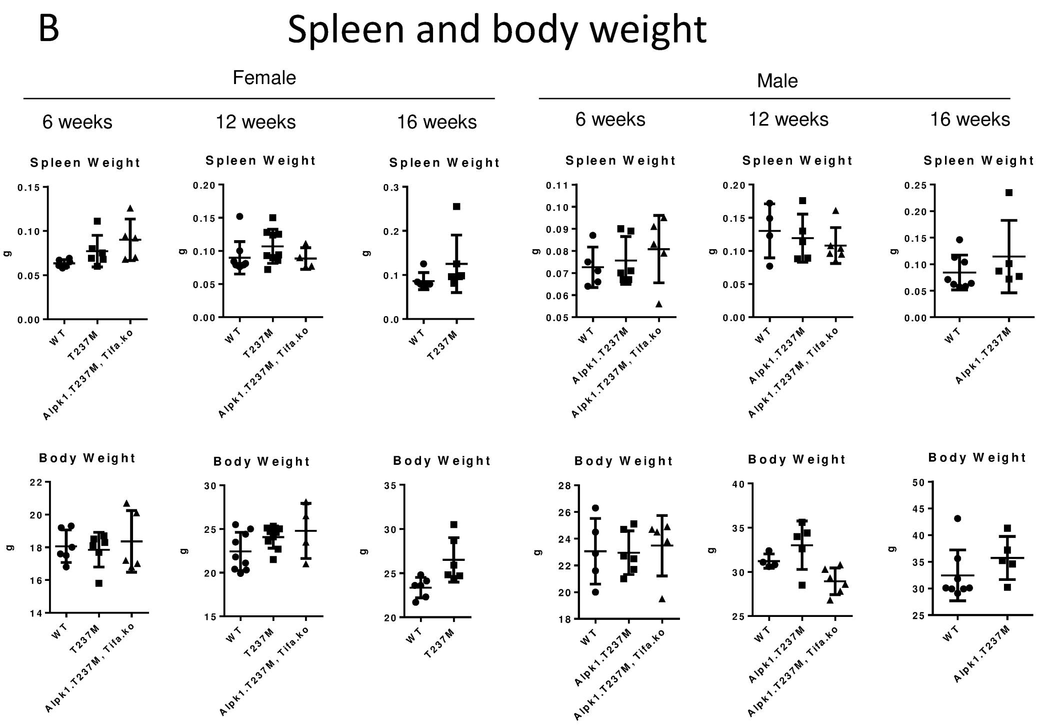

B. Spleen and body weights were determined for mice of 6 weeks, 12 weeks, and 16 weeks of age.

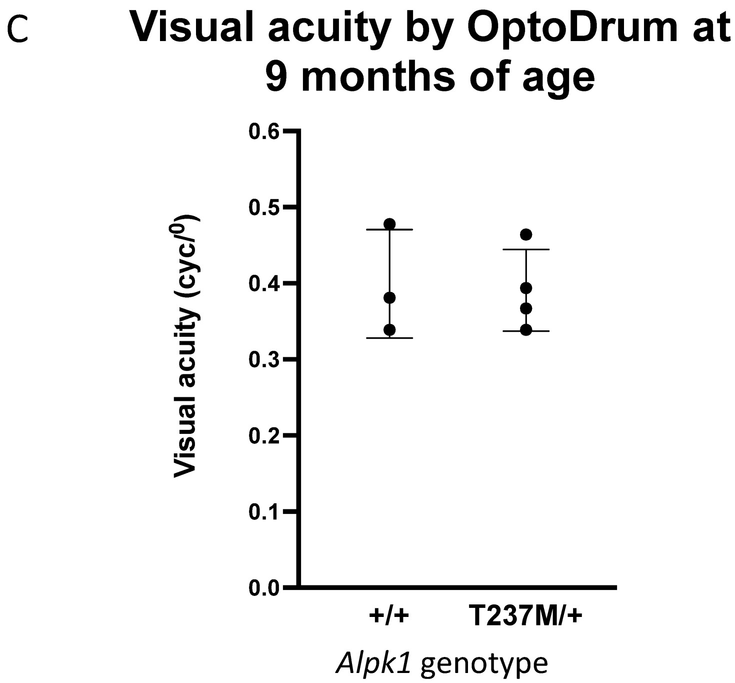

C. Visual acuity in *Alpk1*$^{T237M/WT}$ mice by OptoDrum at 9 months of age.

D Visual function by ERG at 9 months of age

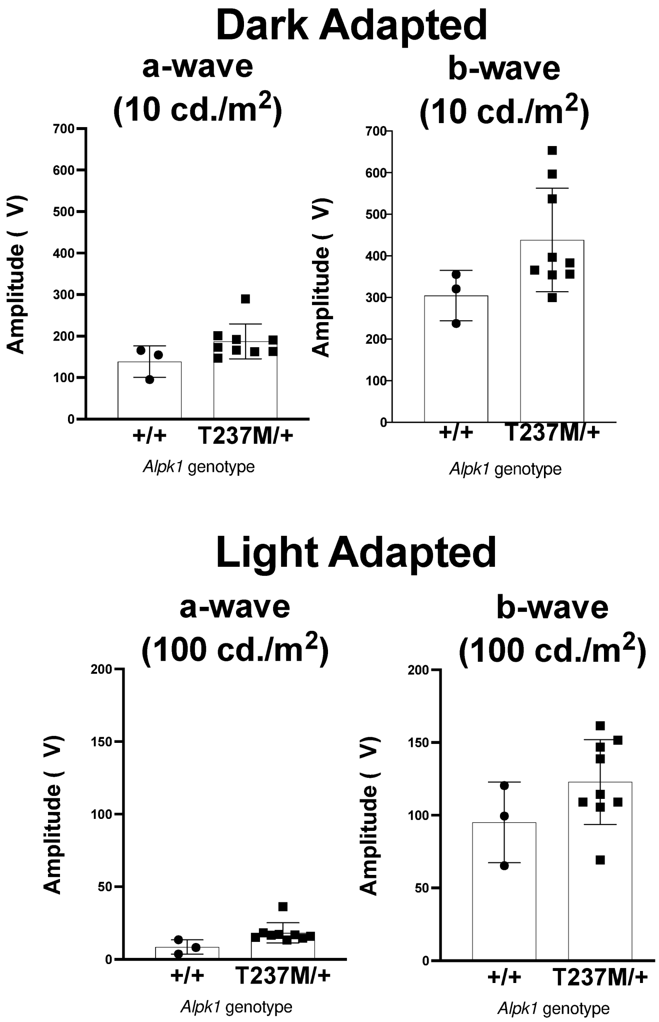

D. Visual function in *Alpk1*$^{T237M/WT}$ mice by ERG at 9 months of age.

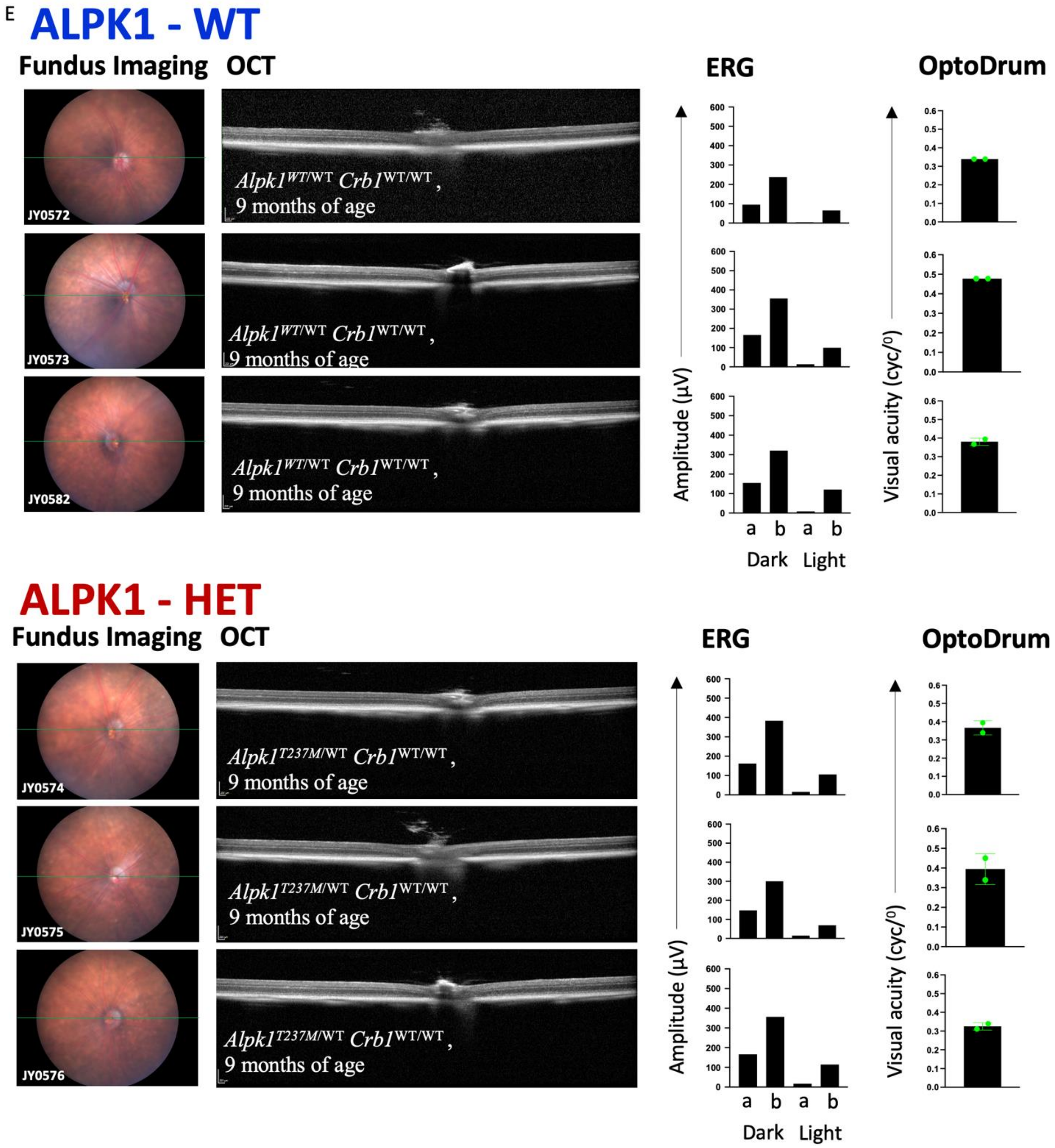
E
ALPK1 - WT
Fundus Imaging
OCT
ERG
OptoDrum
JY0572
Alpk1WT/WT Crb1WT/WT,
9 months of age
JY0573
Alpk1WT/WT Crb1WT/WT,
9 months of age
JY0582
Alpk1WT/WT Crb1WT/WT,
9 months of age
Amplitude (µV)
Visual acuity (cyc/⁰)
a b a b
Dark Light
ALPK1 - HET
Fundus Imaging
OCT
ERG
OptoDrum
JY0574
Alpk1T237M/WT Crb1WT/WT,
9 months of age
JY0575
Alpk1T237M/WT Crb1WT/WT,
9 months of age
JY0576
Alpk1T237M/WT Crb1WT/WT,
9 months of age
Amplitude (µV)
Visual acuity (cyc/⁰)
a b a b
Dark Light

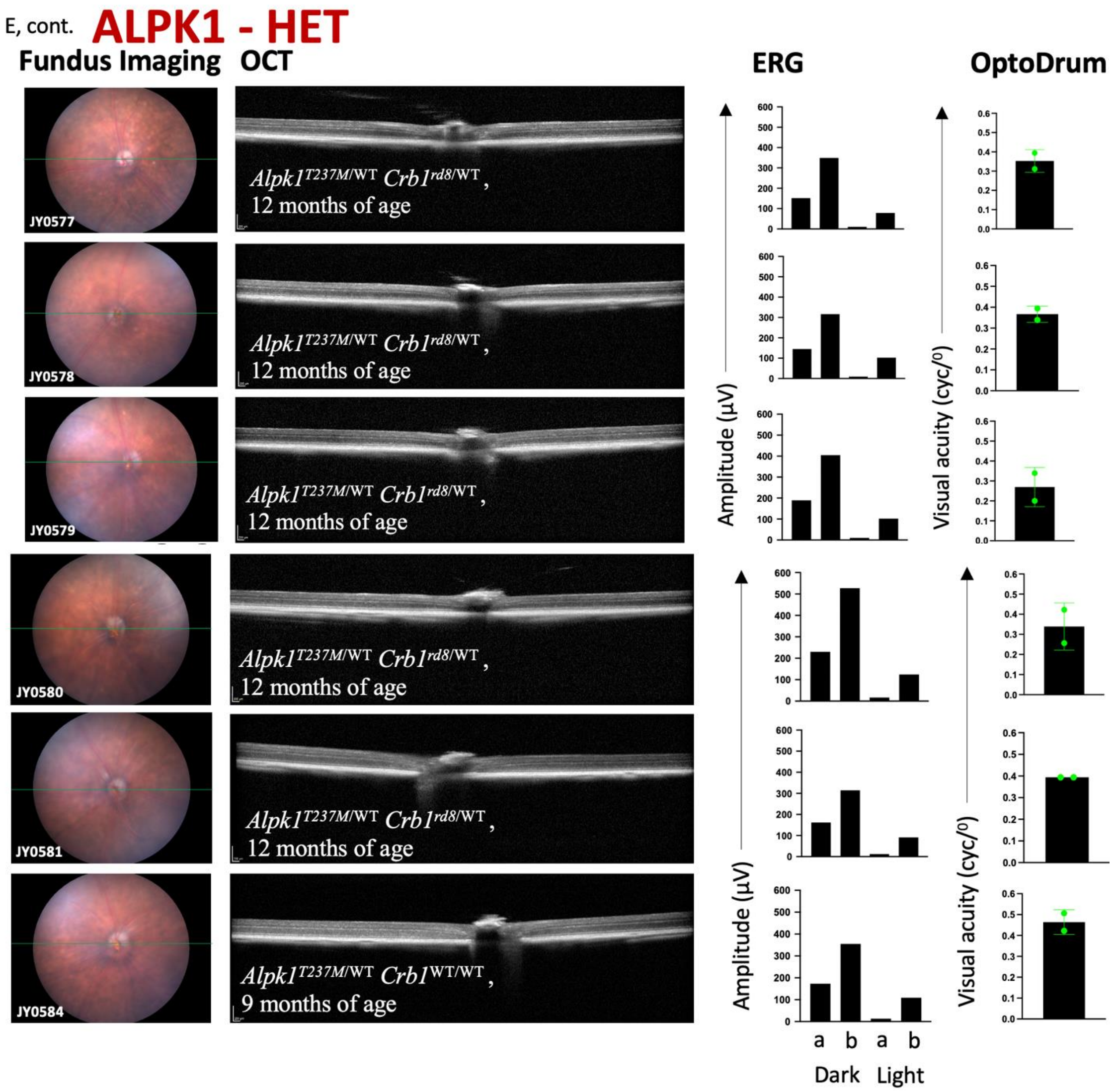

E. Most recent results for fundus imaging, OCT, ERG and OptoDrum evaluations. Age and genotypes as listed.

Supplemental Table 8: List of the 99 differentially expressed inflammatory response genes (GO: 0006954) in whole blood of pre-treatment (n=4) and post-treatment with adalimumab (n=2) patients with ROSAH syndrome.

C3	LRRK2	KLRG1	OGG1
C3AR1	PPARG	TNFRSF1B	ALOX5AP
ICAM1	LACC1	ITGB2	NFAM1
PRKCD	FFAR3	C5AR1	STAT3
IL23R	PIK3CG	HIF1A	CD14
PTX3	SNAP23	LYN	PTGIR
MYD88	NLRC4	IL1RL1	FPR1
TNFAIP3	CXCL8	NLRC3	IL1R1
MAPK14	GBA	GGT1	NLRX1
LOXL3	RELB	IL6R	SLAMF1
FGR	BCL6	DAB2IP	NDST1
IL17RA	CD36	CEBPA	NFE2L2
HCK	ADAM8	FPR2	GGT5
CYBB	ALOX5	PELO	F11R
CXCR6	IL10RB	NLRP12	PTAFR
PRCP	STAP1	TLR1	ORM1
NCF1	NFKB1	EXT1	NOTCH2
VAMP8	SERPINA1	ADGRE2	TLR4
SIRPA	IL1B	PGLYRP1	STAT5B
CCRL2	PLCG2	ZC3H12A	MAPKAPK2
NFKBIZ	PIK3AP1	TLR2	RAC1
GRN	PARP4	PLA2G7	UNC13D
SYK	AREL1	TNIP1	THEMIS2
TICAM2	SIGLEC10	JAK2	AOAH
PTGES	CD44	ADORA2A	

Members of the Undiagnosed Diseases Network

Maria T. Acosta
Margaret Adam
David R. Adams
Justin Alvey
Laura Amendola
Ashley Andrews
Euan A. Ashley
Mahshid S. Azamian
Carlos A. Bacino
Guney Bademci
Ashok Balasubramanyam
Dustin Baldridge
Jim Bale
Michael Bamshad
Deborah Barbouth
Pinar Bayrak-Toydemir
Anita Beck
Alan H. Beggs
Edward Behrens
Gill Bejerano
Jimmy Bennet
Beverly Berg-Rood
Jonathan A. Bernstein
Gerard T. Berry
Anna Bican
Stephanie Bivona
Elizabeth Blue
John Bohnsack
Devon Bonner
Lorenzo Botto
Brenna Boyd
Lauren C. Briere
Elly Brokamp
Gabrielle Brown
Elizabeth A. Burke
Lindsay C. Burrage
Manish J. Butte
Peter Byers
William E. Byrd
John Carey
Olveen Carrasquillo
Thomas Cassini
Ta Chen Peter Chang
Sirisak Chanprasert
Hsiao-Tuan Chao
Gary D. Clark
Terra R. Coakley
Laurel A. Cobban
Joy D. Cogan
Matthew Coggins

F. Sessions Cole
Heather A. Colley
Cynthia M. Cooper
Heidi Cope
William J. Craigen
Andrew B. Crouse
Michael Cunningham
Precilla D'Souza
Hongzheng Dai
Surendra Dasari
Joie Davis
Jyoti G. Dayal
Matthew Deardorff
Esteban C. Dell'Angelica
Katrina Dipple
Daniel Doherty
Naghmeh Dorrani
Argenia L. Doss
Emilie D. Douine
Laura Duncan
Dawn Earl
David J. Eckstein
Lisa T. Emrick
Christine M. Eng
Cecilia Esteves
Marni Falk
Liliana Fernandez
Elizabeth L. Fieg
Paul G. Fisher
Brent L. Fogel
Irman Forghani
William A. Gahl
Ian Glass
Bernadette Gochuico
Rena A. Godfrey
Katie Golden-Grant
Madison P. Goldrich
Alana Grajewski
Irma Gutierrez
Don Hadley
Sihoun Hahn
Rizwan Hamid
Kelly Hassey
Nichole Hayes
Frances High
Anne Hing
Fuki M. Hisama
Ingrid A. Holm
Jason Hom
Martha Horike-Pyne
Alden Huang

Yong Huang
Wendy Introne
Rosario Isasi
Kosuke Izumi
Fariha Jamal
Gail P. Jarvik
Jeffrey Jarvik
Suman Jayadev
Orpa Jean-Marie
Vaidehi Jobanputra
Lefkothea Karaviti
Jennifer Kennedy
Shamika Ketkar
Dana Kiley
Gonench Kilich
Shilpa N. Kobren
Isaac S. Kohane
Jennefer N. Kohler
Deborah Krakow
Donna M. Krasnewich
Elijah Kravets
Susan Korrick
Mary Koziura
Seema R. Lalani
Byron Lam
Christina Lam
Grace L. LaMoure
Brendan C. Lanpher
Ian R. Lanza
Kimberly LeBlanc
Brendan H. Lee
Roy Levitt
Richard A. Lewis
Pengfei Liu
Xue Zhong Liu
Nicola Longo
Sandra K. Loo
Joseph Loscalzo
Richard L. Maas
Ellen F. Macnamara
Calum A. MacRae
Valerie V. Maduro
Bryan C. Mak
May Christine V. Malicdan
Laura A. Mamounas
Teri A. Manolio
Rong Mao
Kenneth Maravilla
Ronit Marom
Gabor Marth
Beth A. Martin

Martin G. Martin
Julian A. Martínez-Agosto
Shruti Marwaha
Jacob McCauley
Allyn McConkie-Rosell
Alexa T. McCray
Elisabeth McGee
Heather Mefford
J. Lawrence Merritt
Matthew Might
Ghayda Mirzaa
Eva Morava
Paolo M. Moretti
Mariko Nakano-Okuno
Stan F. Nelson
John H. Newman
Sarah K. Nicholas
Deborah Nickerson
Shirley Nieves-Rodriguez
Donna Novacic
Devin Oglesbee
James P. Orengo
Laura Pace
Stephen Pak
J. Carl Pallais
Christina GS. Palmer
Jeanette C. Papp
Neil H. Parker
John A. Phillips III
Jennifer E. Posey
Lorraine Potocki
Barbara N. Pusey
Aaron Quinlan
Wendy Raskind
Archana N. Raja
Deepak A. Rao
Anna Raper
Genecee Renteria
Chloe M. Reuter
Lynette Rives
Amy K. Robertson
Lance H. Rodan
Jill A. Rosenfeld
Natalie Rosenwasser
Francis Rossignol
Maura Ruzhnikov
Ralph Sacco
Jacinda B. Sampson
Mario Saporta
C. Ron Scott
Judy Schaechter

Timothy Schedl
Kelly Schoch
Daryl A. Scott
Vandana Shashi
Jimann Shin
Edwin K. Silverman
Janet S. Sinsheimer
Kathy Sisco
Edward C. Smith
Kevin S. Smith
Emily Solem
Lilianna Solnica-Krezel
Ben Solomon
Rebecca C. Spillmann
Joan M. Stoler
Jennifer A. Sullivan
Kathleen Sullivan
Angela Sun
Shirley Sutton
David A. Sweetser
Virginia Sybert
Holly K. Tabor
Amelia L. M. Tan
Queenie K.-G. Tan
Mustafa Tekin
Fred Telischi
Willa Thorson
Cynthia J. Tifft
Camilo Toro
Alyssa A. Tran
Brianna M. Tucker
Tiina K. Urv
Adeline Vanderver
Matt Velinder
Dave Viskochil
Tiphanie P. Vogel
Colleen E. Wahl
Stephanie Wallace
Nicole M. Walley
Melissa Walker
Jennifer Wambach
Jijun Wan
Lee-kai Wang
Michael F. Wangler
Patricia A. Ward
Daniel Wegner
Monika Weisz-Hubshman
Mark Wener
Tara Wenger
Katherine Wesseling Perry
Monte Westerfield

Matthew T. Wheeler
Jordan Whitlock
Lynne A. Wolfe
Kim Worley
Changrui Xiao
Shinya Yamamoto
John Yang
Diane B. Zastrow
Zhe Zhang
Chunli Zhao
Stephan Zuchner
Hugo Bellen
Rachel Mahoney

1. Prasov L, Guan B, Ullah E, Archer SM, Ayres BM, Besirli CG, et al. Novel TMEM98, MFRP, PRSS56 variants in a large United States high hyperopia and nanophthalmos cohort. Sci Rep. 2020;10(1):19986.
2. Marmor M, Fulton A, Holder G, Miyake Y, Brigell M, Bach M. ISCEV Standard for full-field clinical electroretinography (2008 update). Documenta Ophthalmologica. 2009;118(1):69-77.
3. McCulloch DL, Marmor MF, Brigell MG, Hamilton R, Holder GE, Tzekov R, et al. ISCEV Standard for full-field clinical electroretinography (2015 update). Documenta ophthalmologica. 2015;130(1):1-12.
4. Jousse-Joulin S, Nowak E, Cornec D, Brown J, Carr A, Carotti M, et al. Salivary gland ultrasound abnormalities in primary Sjögren's syndrome: consensual US-SG core items definition and reliability. RMD Open. 2017;3(1):e000364.
5. Theander E, Mandl T. Primary Sjögren's syndrome: diagnostic and prognostic value of salivary gland ultrasonography using a simplified scoring system. Arthritis Care Res (Hoboken). 2014;66(7):1102-7.
6. Kuhns DB, Priel DAL, Chu J, Zarember KA. Isolation and Functional Analysis of Human Neutrophils. Curr Protoc Immunol. 2015;111:7.23.1-7..16.
7. Zhou P, She Y, Dong N, Li P, He H, Borio A, et al. Alpha-kinase 1 is a cytosolic innate immune receptor for bacterial ADP-heptose. Nature. 2018;561(7721):122-6.
8. Mattapallil MJ, Wawrousek EF, Chan CC, Zhao H, Roychoudhury J, Ferguson TA, et al. The Rd8 mutation of the Crb1 gene is present in vendor lines of C57BL/6N mice and embryonic stem cells, and confounds ocular induced mutant phenotypes. Invest Ophthalmol Vis Sci. 2012;53(6):2921-7.
9. Chen J, Qian H, Horai R, Chan C-C, Falick Y, Caspi RR. Comparative analysis of induced vs. spontaneous models of autoimmune uveitis targeting the interphotoreceptor retinoid binding protein. PLoS One. 2013;8(8):e72161-e.
10. Groh J, Knöpper K, Arampatzi P, Yuan X, Lößlein L, Saliba A-E, et al. Accumulation of cytotoxic T cells in the aged CNS leads to axon degeneration and contributes to cognitive and motor decline. Nature Aging. 2021;1(4):357-67.
11. Molina DK, DiMaio VJ. Normal organ weights in men: part II-the brain, lungs, liver, spleen, and kidneys. Am J Forensic Med Pathol. 2012;33(4):368-72.

12. Molina DK, DiMaio VJ. Normal Organ Weights in Women: Part II-The Brain, Lungs, Liver, Spleen, and Kidneys. Am J Forensic Med Pathol. 2015;36(3):182-7.

13. Human Protein Atlas [Available from: https://www.proteinatlas.org/ENSG00000073331-ALPK1/immune+cell.

14. The Harriet Lane Handbook: a manual for pediatric house officers. Twenty-first edition. ed. Hughes H, Kahl L, editors. Philadelphia, PA: Elsevier; 2018.
